# IRIDA-ARIES Genomics, a key player in the One Health surveillance of diseases caused by infectious agents in Italy

**DOI:** 10.3389/fpubh.2023.1151568

**Published:** 2023-05-30

**Authors:** Arnold Knijn, Valeria Michelacci, Federica Gigliucci, Rosangela Tozzoli, Paola Chiani, Fabio Minelli, Gaia Scavia, Eleonora Ventola, Stefano Morabito

**Affiliations:** Department of Food Safety, Nutrition and Veterinary Public Health, Istituto Superiore di Sanità, Rome, Italy

**Keywords:** One Health surveillance, foodborne pathogens, genomic, multisectorial, molecular typing workflows, data integration

## Abstract

Pathogen genomics is transforming surveillance of infectious diseases, deepening our understanding of evolution and diffusion of etiological agents, host-pathogen interactions and antimicrobial resistance. This discipline is playing an important role in the development of One Health Surveillance with public health experts of various disciplines integrating methods applied to pathogen research, monitoring, management and prevention of outbreaks. Especially with the notion that foodborne diseases may not be transmitted by food only, the ARIES Genomics project aimed to deliver an Information System for the collection of genomic and epidemiological data to enable genomics-based surveillance of infectious epidemics, foodborne outbreaks and diseases at the animal-human interface. Keeping in mind that the users of the system comprised persons with expertise in a wide variety of domains, the system was expected to be used with a low learning curve directly by the persons target of the analyses' results, keeping the information exchange chains as short as possible. As a result, the IRIDA-ARIES platform (https://irida.iss.it/) provides an intuitive web-based interface for multisectoral data collection and bioinformatic analyses. In practice, the user creates a sample and uploads the Next-generation sequencing reads, then an analysis pipeline is launched automatically performing a series of typing and clustering operations fueling the information flow. Instances of IRIDA-ARIES host the Italian national surveillance system for infections by *Listeria monocytogenes* (Lm) and the surveillance system for infections by Shigatoxin-producing *Escherichia coli* (STEC). As of today, the platform does not provide tools to manage epidemiological investigations but serves as an instrument of aggregation for risk monitoring, capable of triggering alarms on possible critical situations that might go unnoticed otherwise.

## 1. Introduction

The increasing application of Whole Genome Sequencing (WGS) in Public Health surveillance of infectious diseases, offers an excellent opportunity to employ the One Health approach (1) with the integration of both genomic and epidemiological data from different health domains (human, veterinary, food and environment). A One Health implementation allows for not only the precocious detection of outbreaks but also for a better understanding of the role of pathogen reservoirs, evolution and vehicles of transmission, enabling proactive prevention of public health threats.

The Italian National Institute of Health (Istituto Superiore di Sanità, ISS) deployed a genomic surveillance system for foodborne pathogens to shift from the existing typing system mainly based on the analysis of Pulsed Field Gel Electrophoresis (PFGE) profiles. This system is aimed at supporting the epidemiological surveillance of foodborne diseases in the population with specific short and medium/long term goals. The main short term goals were early detection of disperse outbreaks in the community, integration with genomic data from food/environment isolates to discriminate whether a certain food chain and vehicle is implicated or not in an outbreak. Likewise, integration of data and descriptive metadata from human and non-human isolates for source attribution and risk assessment studies were foreseen in the mid/long term to inform and evaluate the adoption of One Health control policies. This is particularly important for STEC control due to the large variety of hosts and sources that may play a role in the spread of infection to the most vulnerable population subgroups. For the purpose, the ARIES Genomics project planned to develop a platform as part of a One Health-Based Conceptual Framework (2, 3) starting with the existing collections of STEC and Lm. To guarantee adequate functionality for users with a wide variety of technical skills, the system had to have a low learning curve, a short chain of information exchange, and a simple but exhaustive user interface. This translated in a combination of essential comprehensive outcomes together with the detailed data available for users with more advanced bioinformatic knowledge. The system's stakeholders include public health professionals with different backgrounds. Laboratories and hospitals upload the data, but they also consume it because feedback of how their data relates to that of other Regions is returned as an incentive to participate to the system. In Italy public healthcare is federated at a regional level, so the platform has the important role to overcome data silos and provide horizontal (Hospitals/Laboratories between each other or Region-Region) as well as vertical (Hospitals/Laboratories-Regions-Central Health Authorities) spread of information. Here, the infrastructure, functionalities and usability of IRIDA-ARIES are described, an web-based platform for multisectoral data collection and bioinformatic analyses in support of a still to be formalized national One Health surveillance.

## 2. Methods

### 2.1. The IRIDA-ARIES platform

**Figure 1:**
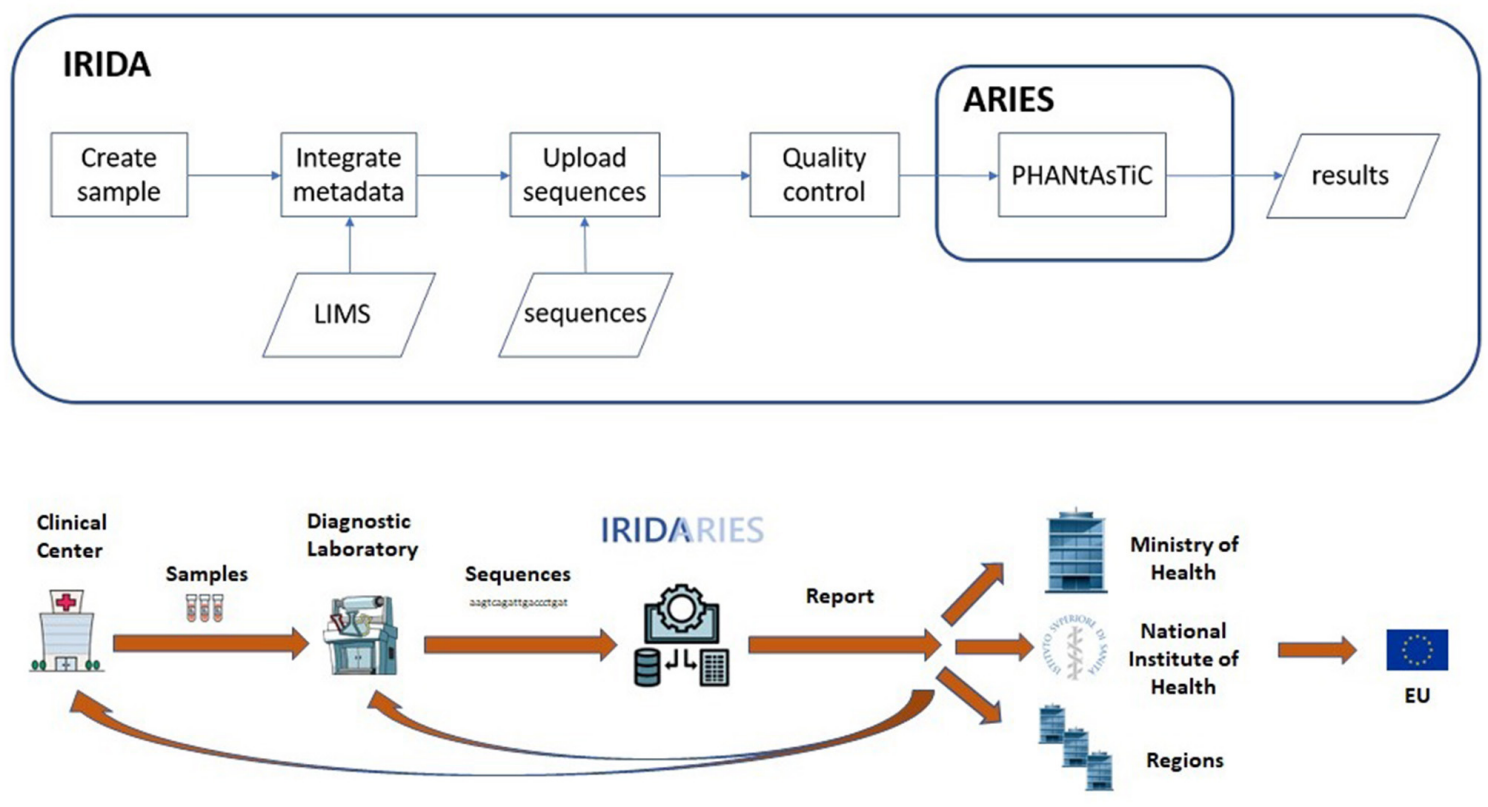
The IRIDA-ARIES platform: when a sample is created, metadata is recovered from both the local molecular and epidemiological information systems. Then, when genomic sequences are uploaded, quality control is performed and consequently the sequences are analyzed by ARIES using the pathogen-specific pipeline PHANtAsTiC. Results are returned to IRIDA and displayed in a specific analysis web page. Schematic design of the IRIDA-ARIES information flow. The flow is triggered at the local level by a clinical center which sends a sample of a case to a diagnostic laboratory (could be in-house) performing the whole genome sequencing. The sequences are uploaded to IRIDA-ARIES and the results are returned to the local level as well as forwarded to the regional and national level. If necessary, the data can be advanced to the European level.

The IRIDA-ARIES genomic surveillance information system is built engaging two open-source platforms: A Galaxy instance (4) implemented as a cluster, ARIES (Advanced Research Infrastructure for Experimentation in genomicS) [preprint (5)] and an IRIDA (Integrated Rapid Infectious Disease Analysis) instance [(6), Figure 1].

The Galaxy Platform is a container for bioinformatic tools sharing a common workflow system, allowing each instance to focus on specific goals through the installation of appropriate tools. Each Galaxy instance is therefore different in function of the aims of the instances' managers. The code of the ARIES instance was installed as a role of the automation platform Ansible from the Galaxy Project (7) and was not changed. Customization of the platform consisted in the development and integration of specific tools and workflows for public health microbiology and molecular epidemiology.

The Galaxy software is not suitable for the collection of samples with genomic and epidemiological data, nor is it possible to implement automation to the analyses. To this means, the open source IRIDA platform fitted the purpose, providing an intuitive web-based interface for the collection of genomics data, utilizing ARIES as a workflow engine for the bioinformatic analyses. In this scenario, IRIDA communicates with ARIES through the latter's unified Applications Programming Interface (API), hiding the ARIES platform from the user, who only interacts with the IRIDA user interface which was translated in Italian.

### 2.2. Integration of heterogeneous genomic data

The IRIDA software package being open source under the Apache License 2.0 was essential for the project because it allowed to fork (copy) the code and adapt the properties of the overall system. The system as a national surveillance platform had to be open to data obtained with various sequencing platforms, not only the mostly used Illumina paired-end reads but also Ion Torrent single reads. Development of bioinformatic tools in pathogen genomics is heavily biased vs. paired-end data. In the Galaxy platform it is not possible to create collections of single reads together with paired-end reads. The IRIDA software was therefore adapted to mask single reads as paired-end and the bioinformatic tools in ARIES were modified correspondently to intercept and elaborate them appropriately. Since this required a matching intervention in ARIES as well as IRIDA breaking functioning of the code, this change could not be opened as a pull request for IRIDA in order to synchronize this feature to the original upstream repository.

Furthermore, since events of infectious diseases launched by European Union Member States on the European Centre for Disease Prevention and Control (ECDC) EpiPulse portal for the European surveillance of infectious diseases (8) frequently only share genomic assemblies (fasta files), the platform had to be able to accommodate and elaborate this type of sequences. Another adaption of both the interface of IRIDA as well as the workflows in ARIES was made. It is therefore possible to create a selection of heterogeneous samples (of both raw and assembled sequences) and launch a workflow using them.

### 2.3. The organization of IRIDA-ARIES

In Italy, healthcare is delegated at the regional level comprising nineteen Regions and two Autonomous Provinces, where each has local health authorities and manages its proper surveillance systems in an independent way, hindering the acquisition of a nation-wide overview (9). To reflect this reality, the organization of the surveillance platform was implemented in a federated way: Regional Projects were created for each pathogen, and the code of the interface was adapted to let these Projects partially share information with a National Project accessible for nation-wide analyses by all regional users (including Competent Authorities) and the Ministry of Health with read-only authorization. Sensitive data present in the Regional Projects is not shared in the National Project in compliance with the General Data Protection Regulation (GDPR, UE n. 2016/679). Upon request of the users, an additional system role was defined, authorized to view results but not to export data. In case of multi-regional clusters, users can see who the members of the other Regions that are involved in the cluster are and contact them directly. Although personalized for the Italian healthcare, this organization is general and may suit a wide variety of contexts.

### 2.4. Information flow

Several customizations have been introduced to automate as much as possible to lower the learning curve for unexperienced users while at the same time providing advanced tools for users with a genomics analysis background. The information flow is data-driven (Figure 1). To contribute to the platform, only two simple operations are required: creation of a sample by providing a unique sample name and upload of the sample's sequence(s). Upon creation of a sample, epidemiological metadata are added or retrieved for data integration from external sources, if available, using the sample name as a key value. Upon upload of the sequence(s), a pathogen-specific workflow is automatically launched performing assembly, typing and clustering elaborations.

After the automated workflow has concluded, an e-mail is sent to Project members containing concise information in function of the pathogen: the end-of-analysis message contains either core genome Multi Locus Sequence Typing (cgMLST) clustering results (whether the sample is part of a cluster, i.e., its genetic profile is similar to those of other samples within a certain cut-off) or variant typing results. In case of a cluster involving more than one Region, the mail is also sent to any other Region involved, to the Ministry of Health and to the ISS to support coordination and outbreak management. A JSON file (Table 1) containing the analytical results is sent attached to the e-mail to allow for automated acquisition of the data by the receiver. Further automation is possible for the user, since the IRIDA batch uploader (10) published by the IRIDA developer team, was adapted to the specific metadata introduced in the IRIDA-ARIES instance and integrated into the system as an FTP service. If necessary, data can be forwarded to the European level.

**Table 1:** Example JSON file containing the analytical results produced by the automatic pipeline PHANtAsTiC for a sample of *Listeria monocytogenes*.

{“coverage”: “153.63”, “read_mean_length”: “139”, “q30_rate”: “0.831981”, “total_bases”: “499125548”, “information_name”: “H_706”, “qc_status”: “Passed”, “qc_messages”: “Passed.”, “serotype_serogroup”: “1/2a,3a”, “serotype_amplicons”: “lmo0737,Prs”, “mlst_ST”: “ST155”, “mlst_CC”: “CC155”, “mlst_lineage”: “II”, “region”: “Lombardia”, “year”: “2022”, “core_genome_schema_size”: 1743, “sample_genes_mapped”: 1729, “Cluster_Id”: “-”}

### 2.5. Molecular typing workflows

All analysis workflows have been designed specifically for the IRIDA-ARIES platform combining both existing as well as in-house developed tools. The workflows used for the automatic elaboration of samples are the most complex. The PHANtAsTiC (Public Health Analysis of Nucleotides through Assembly, Typing and Clustering) workflow [preprint (4)] has been developed to perform a series of pathogen-specific typing tools. All bioinformatic tools that have been integrated into the workflow are listed in Table 2. The assembly phase only applies when raw sequences are uploaded. The sequences are assembled with specific parameters for Ion Torrent or Illumina data and a quality report is generated. In case pre-assembled sequences are provided for samples, this step is skipped. During the typing phase, generic as well as pathogen-specific tools are applied to obtain as much information on the sample as possible. These include serotyping, Multilocus sequence typing (MLST), virulotyping, antimicrobial resistance (AMR) prediction. Aside the molecular typing, a cluster analysis is performed on the distance matrix of the core genome cgMLST profile of each sample with respect to those of all samples present in the platform resulting in a phylogenetic tree. A warning is triggered in case samples are found within a given allele distance threshold which is set at 4 for Lm and 10 for STEC. These values have empirically shown to reflect actual clusters when compared with phylogenetic analyses.

**Table 2:** The bioinformatic tools used in the PHANtAsTiC v2.1 pipeline.

**Phase**	**Step**	**Software/database**	**Version**	**Refences**
Assembly	Trimming	fastp	v0.23.2	(11)
	Assembly Ion Torrent	SPAdes	v3.15	(12)
	Assembly Illumina	INNUca	v4.2.2	(13)
	Assembly quality assessment	QUAST	v.5.0.2	(14)
Typing	Serotyping STEC	BLASTn	v2.11.0	(15)
		Statens Serum Institute database	2022-05-16	(16)
	Serotyping Lm	LisSero	v0.1	(17)
	Multilocus sequence typing (MLST)	mlst	v2.16.1	(18)
		PubMLST typing schemes	7 loci	(19)
	Virulotyping	patho_typing	v0.1	(20)
		Statens Serum Institute database	2022-12-02	(16)
	Shiga toxin subtyping	duk	v0.1	(21)
		Trimmomatic	v0.39	(22)
		SKESA	v2.4	(23)
		SPAdes	v3.15	(12)
		fastq_pair	v1.0	(24)
		MUSCLE	v3.8	(25)
		BLASTn	v2.11.0	(15)
		Statens Serum Institute database	2022-10-18	(16)
	Antimicrobial resistance prediction	ABRIcate	v1.0.1	(26)
		ResFinder	2023	(27)
Clustering	Core genome MLST	chewBBACA	v3.1.2	(28)
		INNUENDO *Escherichia coli* schema	2023	(29)
		Pasteur *Listeria monocytogenes* schema	2023	(30)

The workflow is in its second version since the code has recently been adapted to match the cgMLST typing method performed at a European level by the European Food Safety Authority (EFSA) One Health WGS System (31). In fact, Mentalist (32) has been replaced by chewBBACA (28) as the allele typing method for Lm, while the latter was already used for analyzing STEC samples.

To give users the possibility to further investigate selected samples within the system, several workflows have been added to the platform. These workflows comprise: cgMLST cluster analysis of the previously calculated allele profiles, reference-free Single-Nucleotide Polymorphism analysis using the PopPUNK software v1.1.2 (33), Minimum Spanning Tree analysis of the previously calculated allele profiles with the GrapeTree software v2.2 (34), creation of an HTML summary of the samples with some simple pivot charts, multi virulotyping (calculation of a matrix of samples-virulence genes for the selected samples) for easier comparison between samples, a tool for the creation of an official analysis report in PDF. Expert users can use a workflow to directly export sequences to the ARIES Galaxy instance (5), where a wide variety of genomic and molecular epidemiology bioinformatic tools can be readily used. A copy of the manual of the platform is available as Supplementary material.

### 2.6. Data sharing

Sequences as well as metadata can be easily shared with other systems for further analyses. The IRIDA platform by default features a tool to assist in uploading sequence files to NCBI's Sequence Read Archive. A tool for the export of samples' metadata has been added to the platform. Currently, a collaboration agreement framework is in the process of being finalized, regulating the exchange of human and animal/food/feed Listeriosis data between the National Listeriosis Surveillance Working Group at ISS and the National Reference Laboratory (NRL) for *Listeria monocytogenes* based at the Abruzzo and Molise Veterinary Public Health Institute (IZSAM). Moreover, a tool is under development for the programmatic submission of cgMLST allele profile data to the EFSA One Health WGS system database. Locally, the associated analytical results of STEC data are visible in the web application of the NRL developed for the STEC collection.

### 2.7. Limitations

The customizations to the platform have broken the encapsulation of the two underlying software packages. In fact, masking the heterogeneous data that is shared between them has limited the generality of both systems. Also, ARIES analysis workflows consume data directly from the IRIDA database.

The IRIDA database is implemented on a single server but could be scaled up as a cluster. ARIES is relying on a SLURM (35) cluster for computational capacity and cluster nodes can be easily added if needed using the Ansible automation software. At the moment, ARIES is configured to run all jobs locally, using the file system that is shared between cluster nodes and IRIDA. The installation of a Pulsar server (36) is planned to allow for the execution of jobs on remote High-Performance Computing clusters (HPCs) overcoming the need for a shared file system.

The IRIDA platform is scaled up to four servers for high load deployment, dividing different tasks between them. With this configuration, batch uploads of several thousands of samples have been managed by the system. Currently, no further scaling of the system is possible.

IRIDA-ARIES has to be considered as a component of the applications and protocols to be used in the ecosystem of surveillance, prevention and risk management. Its modular structure and the implemented APIs do allow for the flexible development of personalized interfaces vs. heterogeneous outputs.

## 3. Results

Although the platform is not designed to manage the whole process of surveillance and outbreak management, it comprises features for risk monitoring and is capable of automatically detecting clusters and triggering alarms on possible critical situations. Users are immediately aware of which Regions are involved in the warning and can readily establish connections while keeping information chains short. Regional data is shared to allow for a constantly updated national overview of pathogen diffusion. Feedback is returned to the regional users engaging them to participate actively with their data, creating a virtuous circle avoiding the danger of data silos at the regional level.

Sharing of genomic data facilitates timely detection of clusters and, in general, situations of concern. Furthermore, the exchange with the veterinary public health Institutes (Istituti Zooprofilattici Sperimentali, IZSs) in a One Health view to receive human, animal, food and environmental samples, allows for direct comparison of genomic profiles in order to rapidly exclude possible contamination sources avoiding unnecessary high economic impact and to provide objective arguments to risk management for the timely activation of prevention measures. The exchange of sequence data without its metadata in case of suspect samples would avoid issues with data sharing. Should a situation of suspected outbreak occur, then an integrated data exchange protocol could be activated.

The IRIDA-ARIES platform is currently hosting the Italian national surveillance system for infections by *Listeria monocytogenes* and the local surveillance system for infections by Shiga toxin-producing *Escherichia coli* and counts 71 users, including personnel of the regional Public Health Services. For Listeriosis as of 14/12/2022, a total of 1,453 samples have been uploaded to the platform spanning the period 2002–2022, comprising 1,295 human samples, 61 animal/food/feed historical samples and 97 samples from outbreak events shared through European channels. The platform identified 108 clusters comprising 695 samples (73% of the clusters were composed of 5 or less samples). For STEC as of 14/12/2022, a total of 1,540 samples have been uploaded to the platform spanning the period 1989–2022, comprising 683 human samples, 798 animal/food/feed samples and 59 samples from outbreak events shared through European channels. In this case, 192 clusters have been identified by the platform including 664 samples (90% of the clusters consisted of 5 or less samples). Since PFGE typing was performed only in the presence of an epidemiologically identified suspect cluster and there was no collection of PFGE profiles from the territory, a comparison of cluster detection before and after the switchover is impossible.

The platform has been used to analyze the sequences of 42 STEC and 97 Lm isolates (accessed on 11/11/2022) appended to the information on the events of infectious disease, mainly outbreaks of infections, launched through the ECDC EpiPulse portal or to Urgent Inquiries launched on the former platform Epidemic Intelligence Information System for food- and waterborne diseases (ECDC-FWD-EPIS). The sequences were processed automatically by the platform upon upload and compared with the sequences of all the samples of the same species (for Lm) or serogroup (for STEC) isolated from human cases of disease in Italy already present in the database. This system was used to investigate 30 different events involving STEC strains and 71 involving Lm isolates, allowing to quickly reply on the ECDC FWD system about possible correlations among Italian isolates and those part of ongoing international events.

The platform has proven particularly useful in the investigation of two large outbreaks of Listeriosis that have occurred in Italy in 2022. The presence of two growing clusters, of sequence type 8 and 155 respectively, was noted as evidenced by the platform. Consequently, in particular for the ST155 outbreak, on August 1st 2022 a Working Group was formed by the Ministry of Health, comprising the ISS, the IZSs, the NRL for *Listeria monocytogenes* and the Regions/Autonomous Provinces. The work of this Group supported the epidemiological investigation on the correlation between the clinical cases and the consumption of certain meat products. During the investigation, analysis of the cgMLST profiles allowed for the rapid identification of samples belonging or not to the specific cluster, narrowing the analytical process. The phylogenetic pipelines integrated into the platform have been used by the Working Group for the redaction of the periodic reports as well as autonomously by the regional users themselves. As stated by the Italian undersecretary of the ministry of Health in a parliamentary interrogation: “*The current situation linked to Listeriosis has emerged thanks to the work of the Ministry of Health, through ordinary surveillance and through the IRIDA database of the Istituto Superiore di Sanità, which has made it possible to verify the increase in human cases throughout the national territory*.” (37).

## 4. Discussion

The introduction of the IRIDA-ARIES platform has made the transition from PFGE-based to WGS-based surveillance of listeriosis and STEC infections in Italy smooth, allowing concomitantly to obtain a better overview of the existence of clusters with respect to geographical location as well as to temporal occurrence. In fact, it facilitated the move to a solution joining sample management and user collaboration to combine regional efforts and create a nation-wide view of pathogens' monitoring. Routine sequencing, together with collection of typing data on the territory, has made cluster identification proactive because often the identification of a cluster occurs before the epidemiological suspicion or in the absence of a specific unexpected increase of cases in a given time frame and area. Moreover, the analytical results are shared in real-time to stakeholders in various information systems without being copied by hand, speeding up the process and eliminating repeated tasks and possible errors during transcription. By applying genomics-based surveillance to infectious diseases, One Health practitioners can identify the specific genetic makeup of a pathogen, providing information on the hazard characterization and use this information to predict its potential for spread and to develop targeted interventions. The possibility to upload pre-assembled sequences from European outbreaks originating from both human (ECDC) and animal/food/feed (EFSA) concerning food- and waterborne diseases and zoonoses for a direct comparison with national samples, allows to integrate the Italian surveillance of foodborne diseases within an international One Health perspective. The objective is to align the typing workflows for each pathogen in collaboration with these European Agencies to obtain compatible results that can be readily exchanged.

In 2019, a face-to-face course was organized for the future regional users of the system. The feedback has been very positive, before the end of the course many participants had become confident with the system and acquired the ability to use most applications of the platform. Also, several requests from the participants could be readily implemented. A helpdesk has been set up to assist users running into problems. Now that the restrictions due to the COVID-19 pandemic have been largely lifted, an annual in-person meeting of the *Listeria* network has been foreseen, so the regional users get to know their counterparts from the other Regions, facilitating contacts in case of inter-regional clusters.

The system has been well-accepted by all different types of users because it has proven intuitive enough for those without specific computer skills, while yet powerful for the needs of the users with advanced bioinformatic experience. Although submission of data is on a voluntary basis in Italy, the system is now used by the majority of the Regions. Several clusters persistent in time and/or location have been highlighted by the system, indicating the platform as a powerful tool in support of future preparedness of early detection of food safety risks.

Integration of human genomic data with samples originating from other One Health domains allows the platform to act as a key player in the surveillance of diseases caused by infectious agents in Italy. Not only in Italy though, the platform has been designed as multi-language and can readily be used in any (inter-)national context upon addition of a language dictionary. Issues with data sharing include data ownership, privacy regulations and legal considerations and have been tackled on several levels. The collection of the data has been approved by the Data Protection Officer of the ISS. The Regions remain owners of the genomic data they provide, their sequences cannot be accessed by others but only used in aggregated analyses.

The platform has been used for STEC and Lm because the ISS already collected data for these pathogens and therefore the expertise for analyzing these genomes had been previously acquired. The surveillance of other pathogens could be implemented without much effort since the bioinformatic tools of the platform can be flexibly adapted. An IRIDA-ARIES instance named ICoGen (Italian COVID Genomics), is actually in use at the ISS for the national surveillance of genomic variants of the SARS-CoV-2 virus.

In the mid/long term, the IRIDA-ARIES infrastructure is meant to become the national platform for the genomic surveillance of infectious diseases. In this respect, the established networks providing data on Lm and STEC isolates from the different Italian regions, will be the starting point for expanding and consolidating the data providers' network for other foodborne infections. The analytical metadata of the sequenced strains will represent the central elements for the prompt identification of outbreak events as well as for source attribution and exposure risk assessment. Further development will focus on the integration of the platform as a component of an overall infrastructure for the surveillance and management of infectious diseases. The hope is that IRIDA-ARIES through the establishment of an inclusive cross-sector network will serve as a basis and stimulus for the creation of a national systemic approach enabling source attribution studies such as those carried out in the DiSCoVeR project (38) and possibly adapting solutions already implemented and new tools for surveillance and risk assessment still under development in projects such as COHESIVE (39) which is part of the One Health European Joint Program (40). Furthermore, the next step will include a FAIRification process of the produced datasets to enhance machine findability, accessibility, interoperability and reusability (41). The latter will be crucial for the integration of the heterogenous data collected during the various levels of a One Health surveillance and risk assessment infrastructure. FAIR principles for data and software are generally applicable, but need to be extended in order to address the processual nature of workflows, which will pave the way for standardized trustable data with the added value of being ready for secondary data reuse and exploitation by third parties (42).

## Data availability statement

The datasets presented in this study can be found in online repositories. The names of the repository/repositories can be found below: IRIDA-ARIES: https://github.com/aknijn/irida PHANtAsTiC: https://github.com/aknijn/phantastic-galaxy.

## Collaborator group members

### European Union Reference Laboratory for *Escherichia coli*

Arianna Boni, Paola Chiani, Guendalina Fornari Luswergh, Federica Gigliucci, Arnold Knijn, Valeria Michelacci, Fabio Minelli, Margherita Montalbano Di Filippo, Stefano Morabito, Rosangela Tozzoli.

### National Listeriosis Surveillance Working Group

Gianni Ciccaglioni, Alfonsina Fiore, Antonietta Gattuso, Marco Francesco Ortoffi.

### IRIDA-ARIES user group STEC

Stefano Bilei, Paola Chiani, Giuliano Garofolo, Federica Gigliucci, Arnold Knijn, Valeria Michelacci, Stefano Morabito, Antonio Parisi, Gaia Scavia, Rosangela Tozzoli, Eleonora Ventola.

### IRIDA-ARIES user group Listeriosis

Richard Aschbacher, Stefano Bilei, Giuliana Blasi, Teresa Bossù, Daniela Cecconi, Lisa Chenal, Maria Chironna, Veronica Cibin, Gianni Ciccaglioni, Valeria Cosma, Mauro Cravero, Michele d'Errico, Maria Laura De Marchis, Paola De Santis, Lucia Decastelli, Federica Ferraro, Alfonsina Fiore, Alessia Franco, Laura Gasperetti, Antonietta Gattuso, Elisabetta Giacobazzi, Claudio Giacomazzi, Federica Gigliucci, Maria Gori, Arnold Knijn, Regione Lazio Seresmi, Daniela Loconsole, Daniela Lombardi, Sarah Lovari, Paola Marconi, Elisa Masi, Riccardo Mazzocca, Elena Mazzolini, Valeria Michelacci, Onofrio Mongelli, Stefano Morabito, Marina Morganti, Ornella Moro, Marco Francesco Ortoffi, Elisabetta Pagani, Barbara Palombo, Antonio Parisi, Stefano Pongolini, Monica Pitti, Erika Scaltriti, Gaia Scavia, Cristina Schellenberger, Elisabetta Tanzi, Rosangela Tozzoli, Francesco Vairo, Eleonora Ventola, Teresa Zaccaria, Cristina Zappetti, Salvatore Zingale.

### Italian Registry of Hemolytic Uremic Syndrome

Francesca Becherucci, Elisa Benetti, Cristina Bertulli, Maurizio Brigotti, Milena Brugnara, Roberta Camilla, Valentina Capone, Roberto Chimez, Maria Chironna, Ciro Corrado, Alessandra Gianviti, Mario Giordano, Arnold Knijn, Claudio La Scola, Daniela Loconsole, Ilse Maria Ratsch, Laura Massella, Marco Materassi, Valeria Michelacci, Fabio Minelli, Mattia Parolin, Andrea Pasini, Carmine Pecoraro, Marco Pennesi, Licia Peruzzi, Fabrizio Pugliese, Gaia Scavia, Rosangela Tozzoli, Antonella Trivelli, Eleonora Ventola, Enrico Verrina, Enrico Vidal, Marina Vivarelli.

## Collaborator group members contributions

The members of the European Union Reference Laboratory for *Escherichia coli* collected STEC samples and sequenced the DNA for molecular typing. The members of the National Listeriosis Surveillance Working Group collected Listeriosis samples and sequenced the DNA for molecular typing. The members of the IRIDA-ARIES user group STEC collected STEC samples and sequenced the DNA for molecular typing. The members of the IRIDA-ARIES user group Listeriosis collected the samples and sequenced the DNA for molecular typing. The members of the Italian Registry of Hemolytic Uremic Syndrome collected STEC samples.

## Author contributions

AK and SM were responsible for the concept and design of the study, interpretation of results, writing, and critical review of the manuscript. AK was responsible for the design and development of the IRIDA-ARIES platform. AK, VM, FG, and SM were responsible for the design of the bioinformatic workflows. RT, PC, and FM were responsible for the collection and curation of data from the STEC registry. GS and EV were responsible for the collection and curation of the epidemiologic data of the Italian Registry of Hemolytic Uremic Syndrome. All authors contributed to the article and approved the submitted version.
