# Erratum: IRIDA-ARIES Genomics, a key player in the One Health surveillance of diseases caused by infectious agents in Italy

**DOI:** 10.3389/fpubh.2023.1276201

**Published:** 2023-09-05

**Authors:** Frontiers Production Office

**Affiliations:** Frontiers Media SA, Lausanne, Switzerland

**Keywords:** One Health surveillance, foodborne pathogens, genomic, multisectorial, molecular typing workflows, data integration

Due to a production error, the consortia were incorrectly formatted.

The author byline was previously:

“Arnold Knijn, Valeria Michelacci, Federica Gigliucci, Rosangela Tozzoli, Paola Chiani, Fabio Minelli, Gaia Scavia, Eleonora Ventola, Stefano Morabito, National Listeriosis Surveillance Working Group, IRIDA-ARIES User Group STEC, IRIDA-ARIES User Group Listeriosis and Italian Registry of Hemolytic Uremic Syndrome on behalf of European Union Reference Laboratory for *Escherichia coli*”

The corrected author byline appears below:

“Arnold Knijn, Valeria Michelacci, Federica Gigliucci, Rosangela Tozzoli, Paola Chiani, Fabio Minelli, Gaia Scavia, Eleonora Ventola and Stefano Morabito on behalf of National Listeriosis Surveillance Working Group, IRIDA-ARIES User Group STEC, IRIDA-ARIES User Group Listeriosis, Italian Registry of Hemolytic Uremic Syndrome, European Union Reference Laboratory for *Escherichia coli*”

The author and consortia contribution statements were previously listed together. They are now listed separately, and the corrected statements appear below:

## Collaborator group members contributions

“The members of the European Union Reference Laboratory for *Escherichia coli* collected STEC samples and sequenced the DNA for molecular typing. The members of the National Listeriosis Surveillance Working Group collected Listeriosis samples and sequenced the DNA for molecular typing. The members of the IRIDA-ARIES user group STEC collected STEC samples and sequenced the DNA for molecular typing. The members of the IRIDA-ARIES user group Listeriosis collected the samples and sequenced the DNA for molecular typing. The members of the Italian Registry of Hemolytic Uremic Syndrome collected STEC samples.”

## Author contributions

“AK and SM were responsible for the concept and design of the study, interpretation of results, writing, and critical review of the manuscript. AK was responsible for the design and development of the IRIDA-ARIES platform. AK, VM, FG, and SM were responsible for the design of the bioinformatic workflows. RT, PC, and FM were responsible for the collection and curation of data from the STEC registry. GS and EV were responsible for the collection and curation of the epidemiologic data of the Italian Registry of Hemolytic Uremic Syndrome. All authors contributed to the article and approved the submitted version.”

The publisher apologizes for this mistake. The original article has been updated.

