# Supplementary material for: IRIDA-ARIES Genomics, a key player in the One Health surveillance of diseases caused by infectious agents in Italy
Source: Front Public Health. 2023 May 30;11:1151568. doi: 10.3389/fpubh.2023.1151568 (PMC10289303; doi:10.3389/fpubh.2023.1151568)

# Manual of the platform

IRIDARIES

v2.2 02/05/2023

# Istituto Superiore di Sanità

Department of Food safety, nutrition and veterinary public health

Microbiological safety of food and foodborne diseases Unit – One Health MTA

# Sommario

**Piattaforma per la raccolta e l’analisi dei dati NGS** ............ 4

**Accesso alla piattaforma** ............ 4

**Accesso ai Progetti** ............ **Errore. Il segnalibro non è definito.**

**Inserire nuovi campioni nel Progetto della Regione** ............ **Errore. Il segnalibro non è definito.**

**Il Progetto della Sorveglianza** ............ **Errore. Il segnalibro non è definito.**

**Centro Analisi** ............ 17

# Platform for the collection and analysis of NGS data

## Access to the platform

IRIDA-ARIES can be accessed at the following address, https://irida.iss.it/

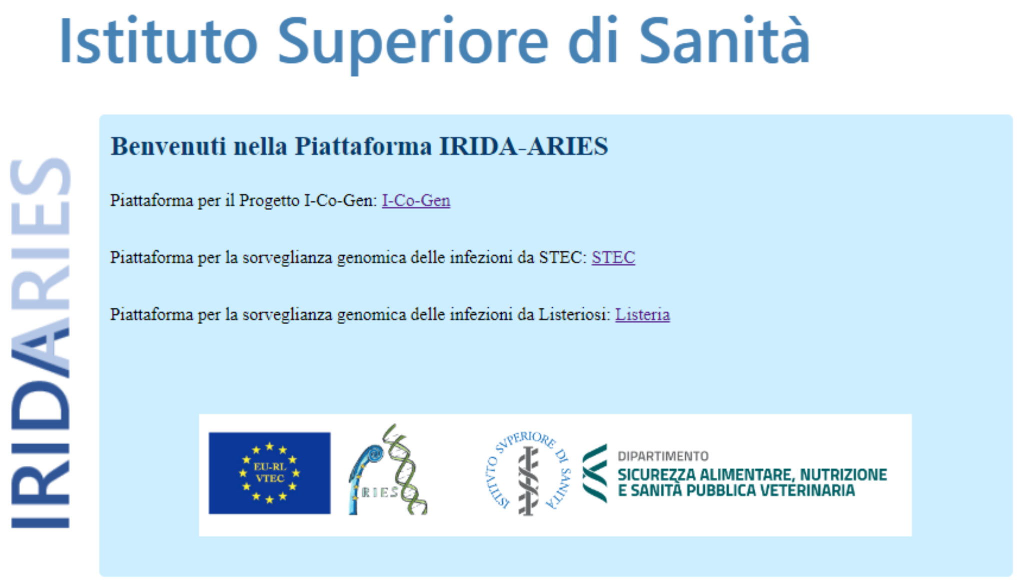


From here the user can select the specific platform to access.

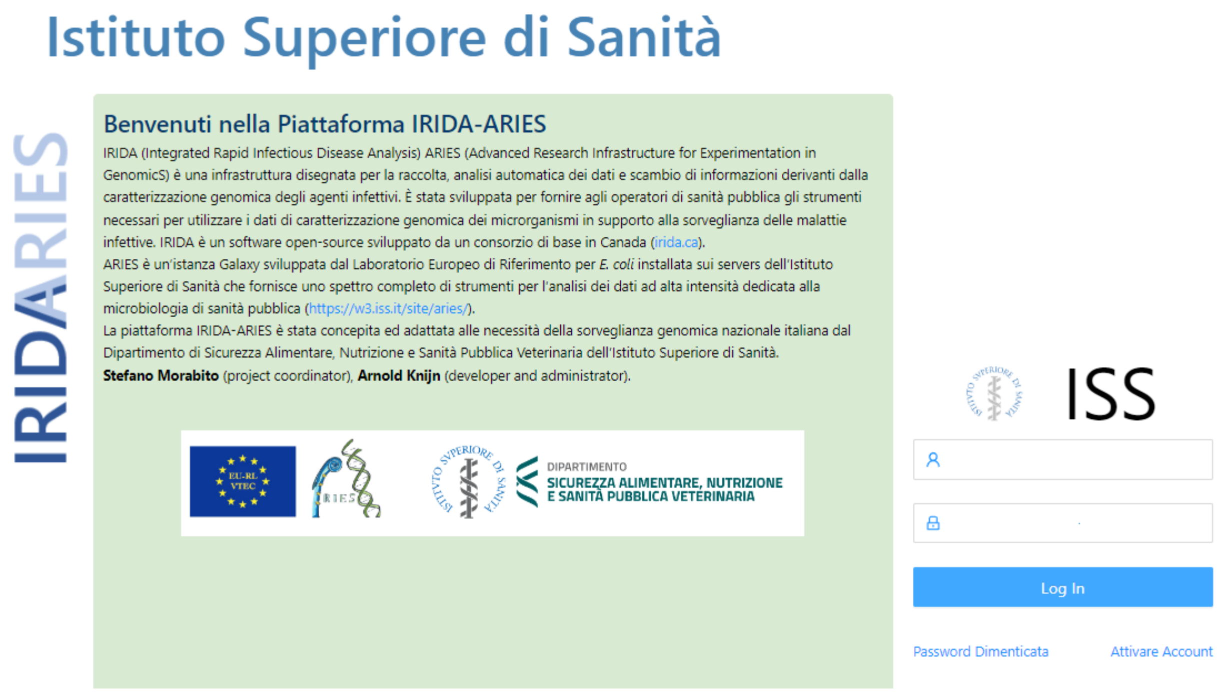


The username is generally the institutional e-mail address and the password was set by the user upon activation of the account.

After logging in, the user is directed to the main page.

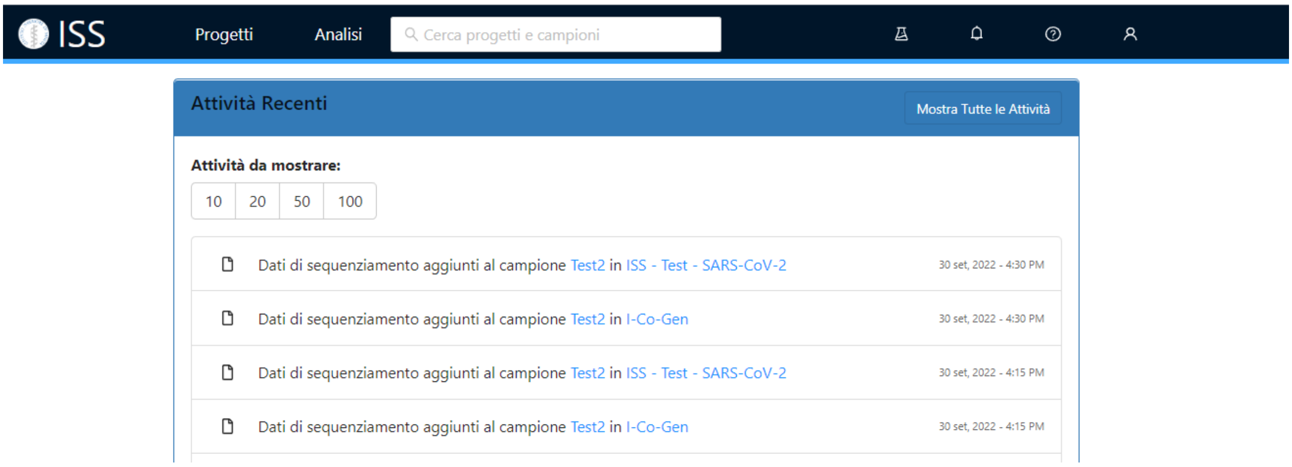


The main page is composed at the top by a dark ribbon which on the left side contains the ISS logo which redirects to the main page and the two menus **Progetti** (Projects) and **Analisi** (Analyses).

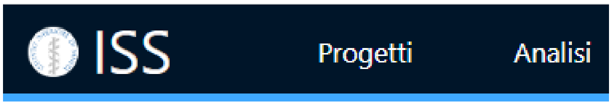


Instead, on the right side there are respectively the global search box, the Analysis Center icon, the announcements menu, the **Help** menu and the **Account** menu.

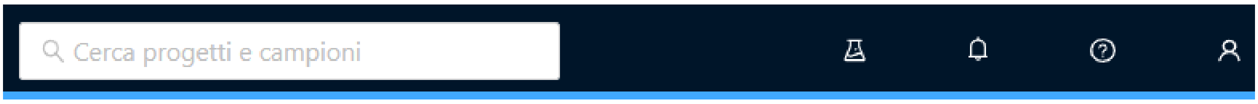


In the central part of the page, recent activities are shown.

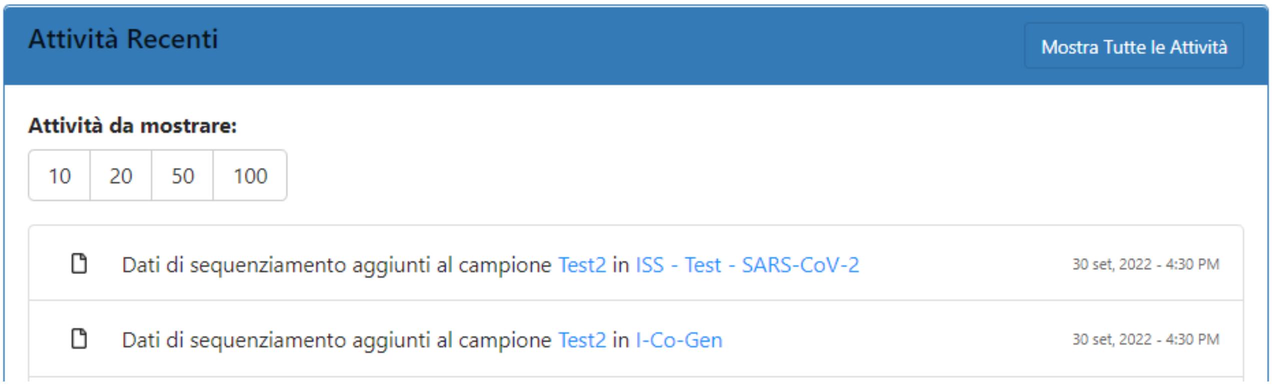

# Access to the Projects

Opening the menu **Progetti** (Projects), the **Propri Progetti** (Own Projects) item is displayed which, once selected, will redirect to the page that will list the user's Projects with some information.

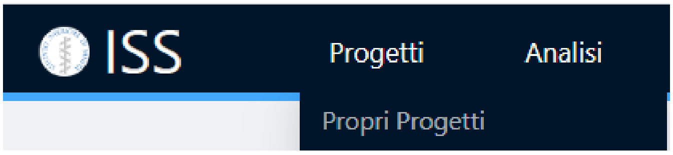


The user will see two Projects: the Project of the belonging Region and the general surveillance Project with national data.

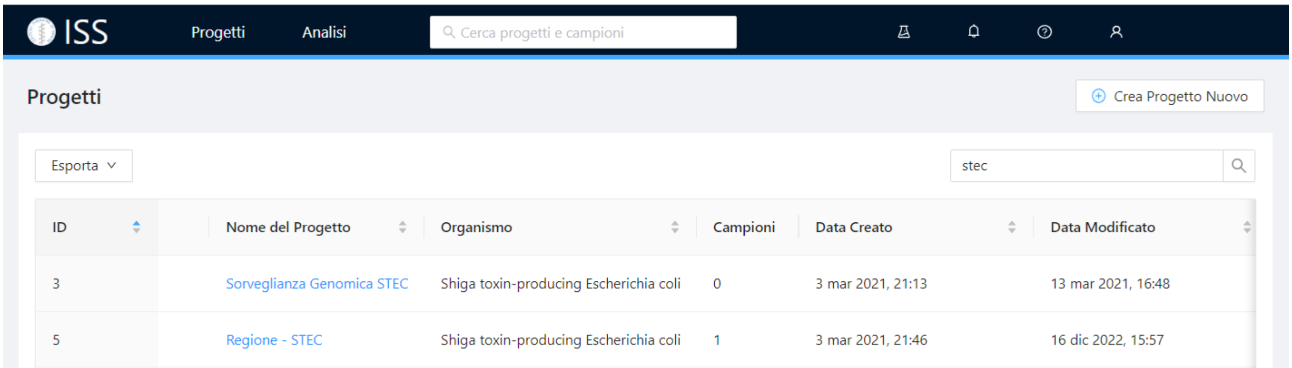


By clicking on the name of the Project of the Region, regional data is accessed.

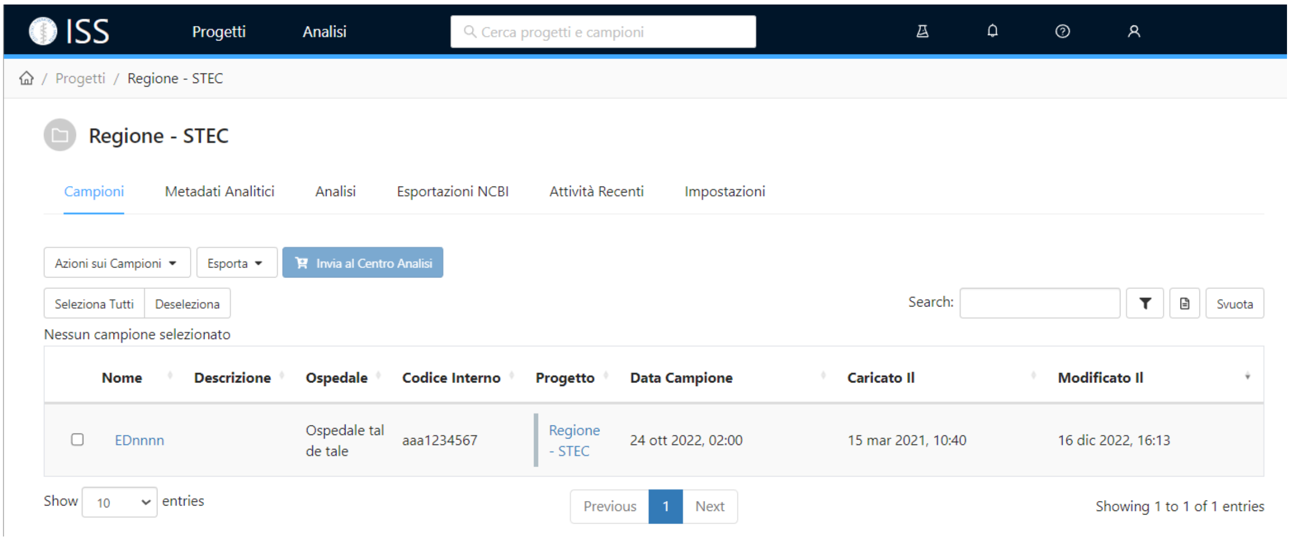


The Project page of the Region contains a menu with six items to access as many sections.

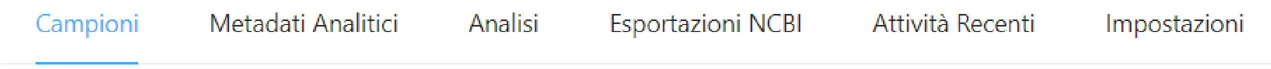


In the section **Campioni** (Samples) samples of the project with some information are listed. Also, there are two menus **Azioni sui Campioni** (Actions on Samples) and **Esporta** (Export) and the button **Invia al Centro Analisi** (Send to the Analysis Centre).

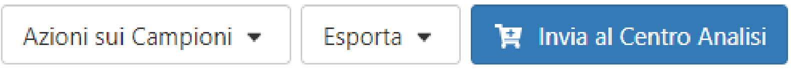


The actions that can be performed on samples are **Unisci** (Unite), **Condividi** (Share) and **Rimuovi** (Remove). Finally, sample metadata can be imported from files and new samples added.

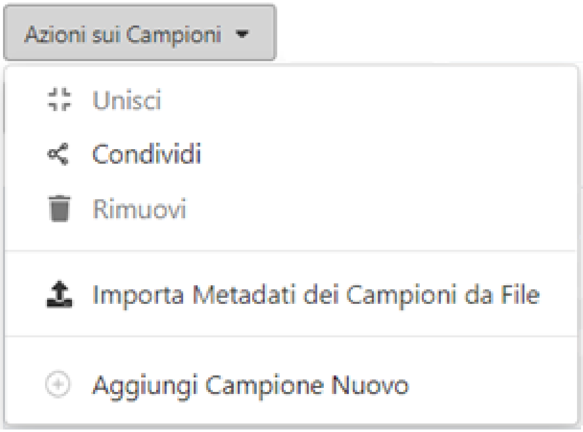


In the menu **Esporta** (Export), samples can be downloaded and exported in Excel or a comma-separated csv file.

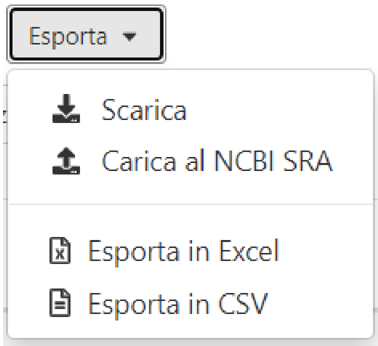


By selecting samples, they can be sent to the Analysis Center to be inserted into the analytical pipelines using the appropriate button.

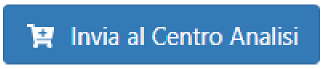


With the buttons **Seleziona Tutti** (Select All) and **Deseleziona** (Unselect) sample selection can be facilitated.

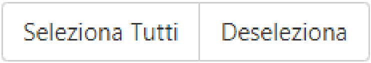


here are various ways to search for samples: by typing in the text box, the list of samples will immediately be filtered by the name of the sample.

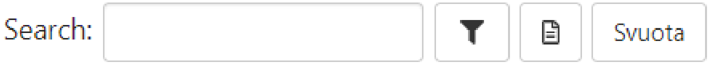


By clicking on the funnel button instead, a window will open with the possibility of setting filters on various columns. Finally, to simplify the selection of large numbers of samples from projects, a text

file containing the names of the samples to be analyzed can be used, with each name on one line. By clicking on the button with the document icon and selecting this file, the samples with matching names will be filtered and selected. With the **Svuota** (Clear) button all filters are reset.

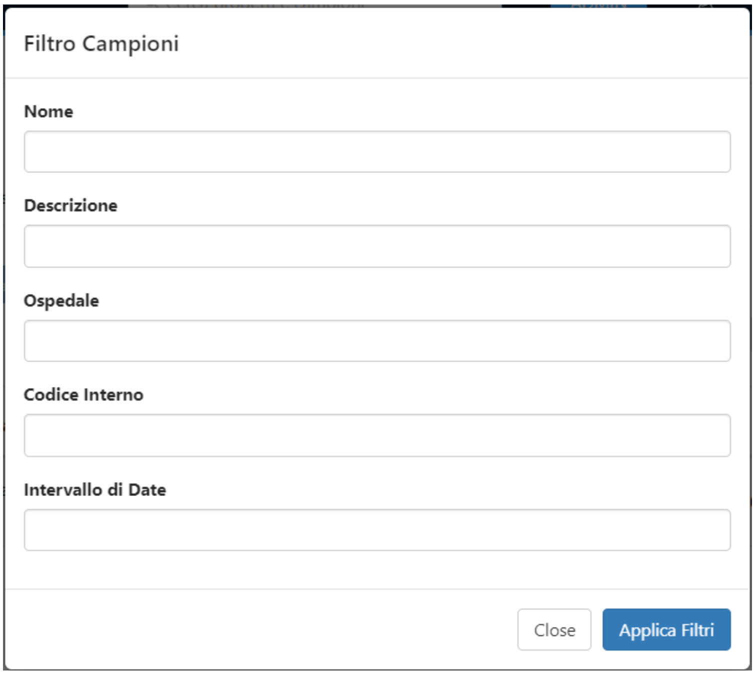


In the section **Metadati Analitici** (Analytical Metadata), the list of samples is enriched with the metadata obtained by automatic analysis upon uploading the sequences with the pipeline PHANtAsTiC (*Public Health Analysis of Nucleotides through Assembly, Typing and Clustering*) or RECoVERY (*REconstruction of CoronaVirus gEnomes & Rapid analYsis*) according to the organism under investigation.

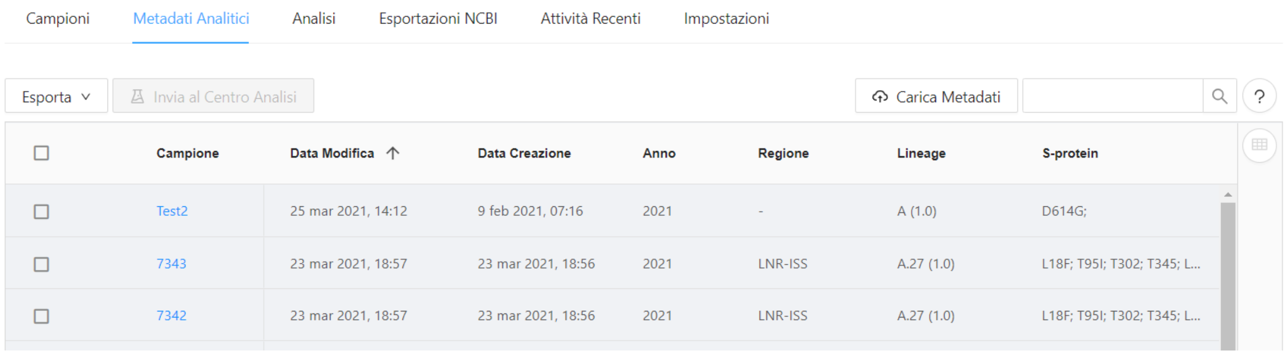


In this case, the menu **Esporta** (Export) only provides for exporting data as Excel and CSV.

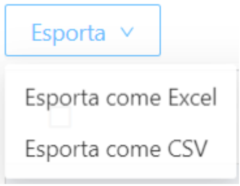

The button **Importa Metadati dei Campioni da File** (Import Sample Metadata from a File) opens a page where an Excel file can be loaded to automatically update the descriptive fields of the samples.

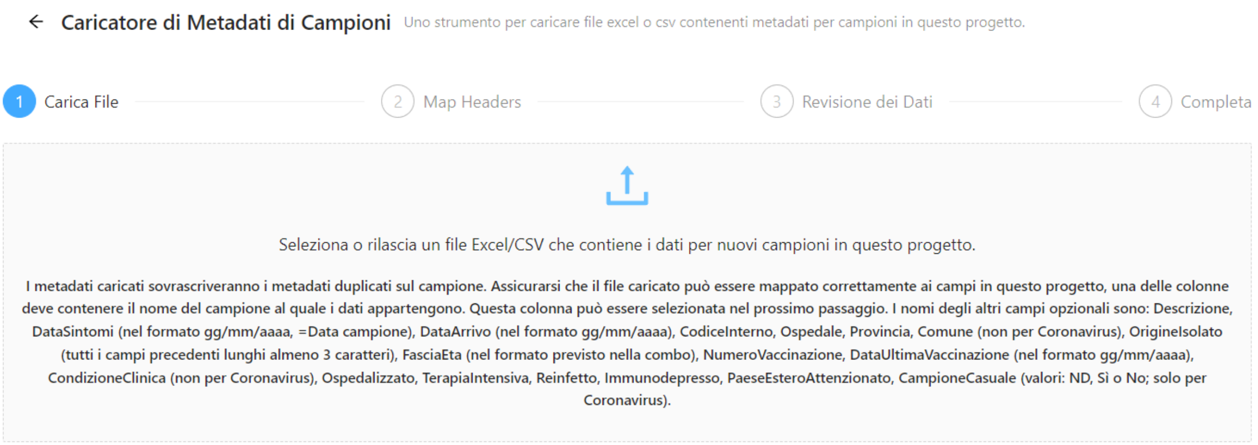


The text box allows to search all the fields of the samples table, but there is also the possibility of performing more specific searches. Moving the mouse pointer over each column header, a three horizontal lines icon will appear to the right. By clicking on it, a box opens where it is possible to set filters on the same column..

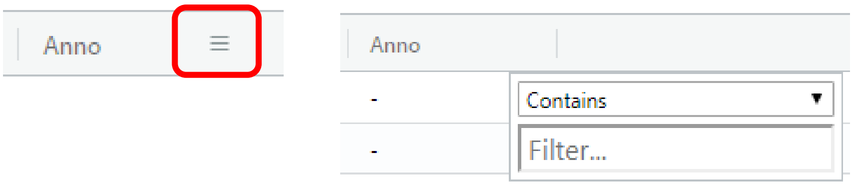


Pressing the round button with the question mark activates a short illustration of all the options on this page. While the round button with the two columns allows to display/hide the columns of the table.

By pressing the round icon with the question mark, a tour starts with an explanation of the functionality of the analytical data table.

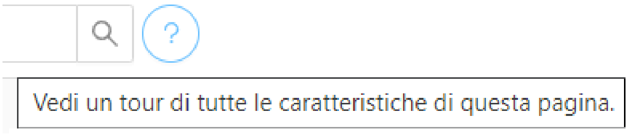


In the section **Analisi** (Analysis), all analyses performed and shared with the project are listed with the possibility to perform searches.

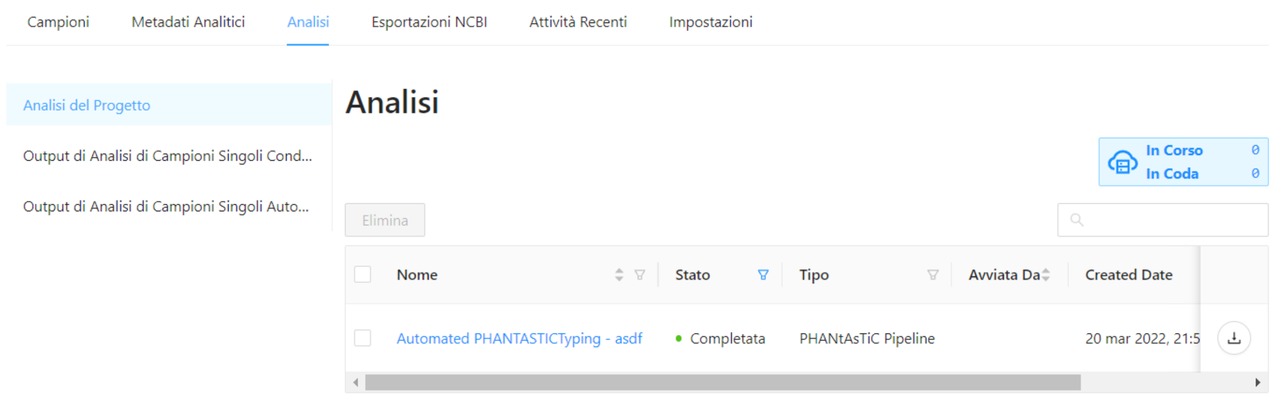


Choosing the option **Output di Analisi di Campioni Singoli Condivisi** (Output of Shared Single Sample Analyses) or alternatively **Output di Analisi di Campioni Singoli Automatizzati**, (Output of Automated Single Sample Analyses) the possibility exists to download the output files of the analyses performed using the button **Download**.

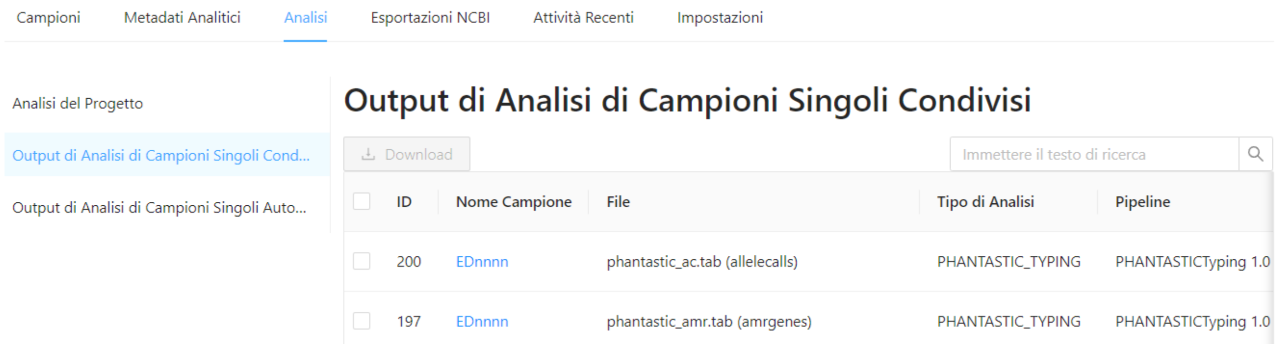


The section **Esportazioni NCBI** (NCBI Export) lists the samples sent to NCBI.

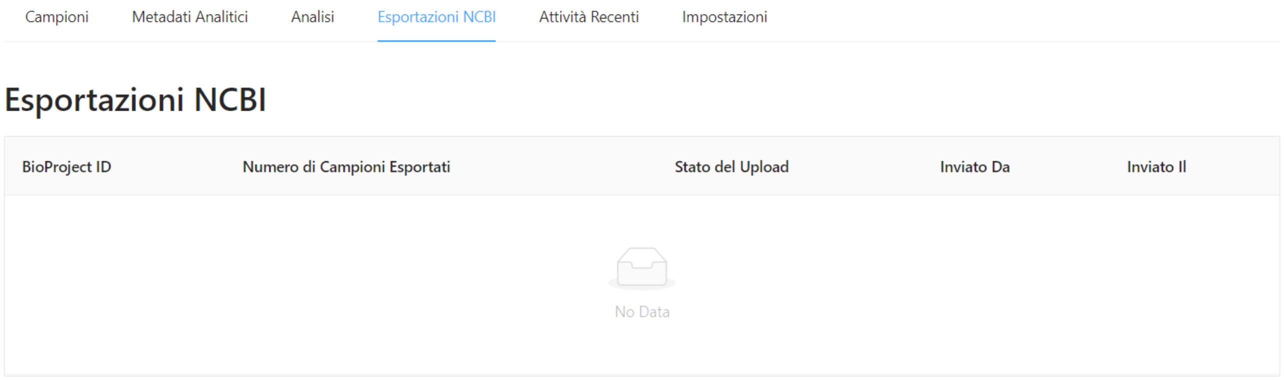


The section **Attività Recenti** (Recent Activities) reports the latest activities concerning the project.

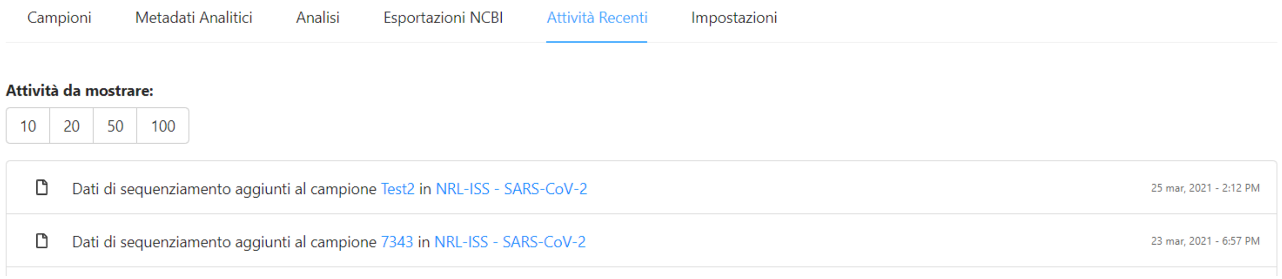

The section **Impostazioni** (Configuration) reports details of the project.

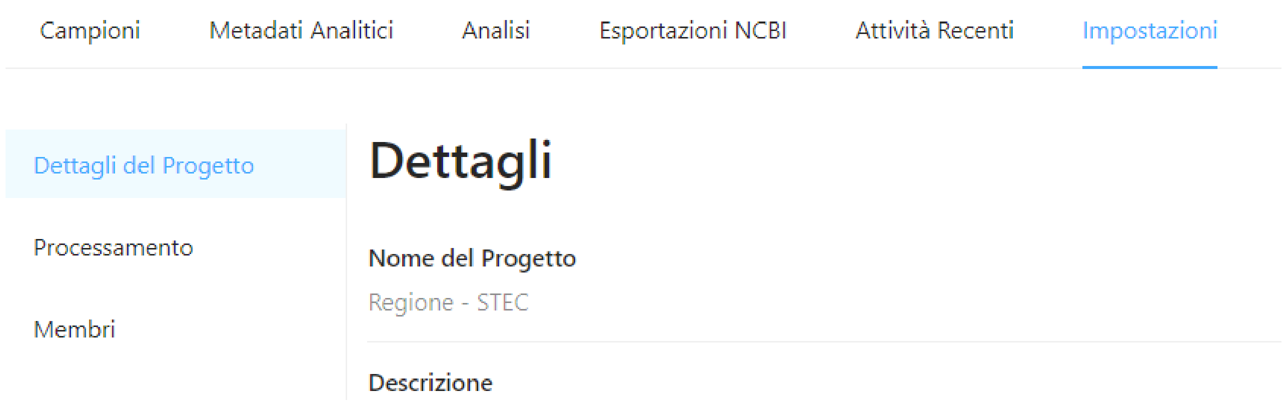


In the subsection **Processamento**, which pipeline is automatically executed when a new sequence is loaded and the Coverage values set for the project are shown.

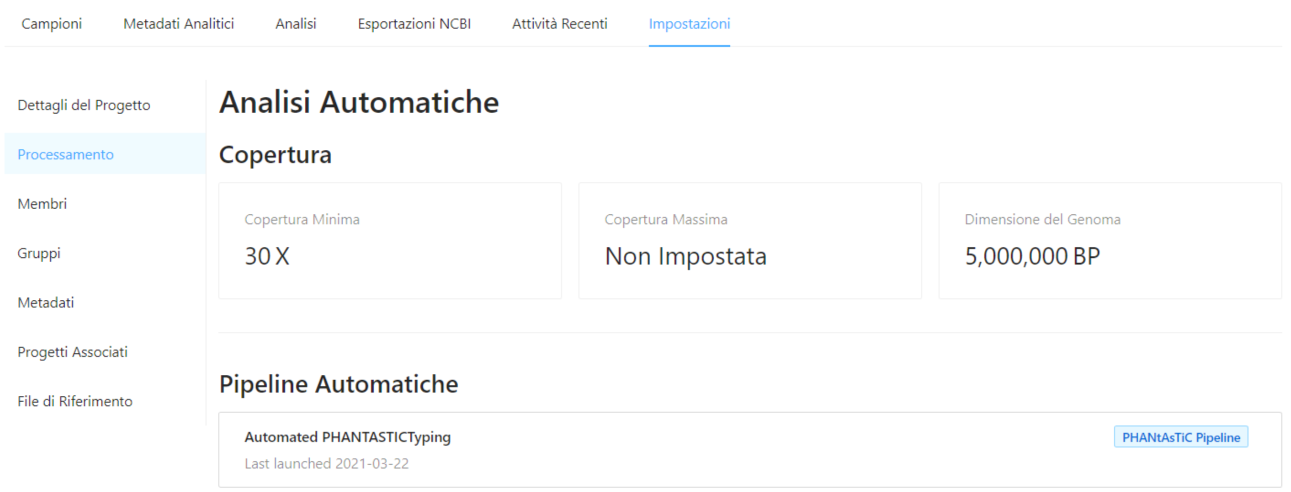


Instead, by pressing the **Membri** (Members) button, the people connected to the project are listed.

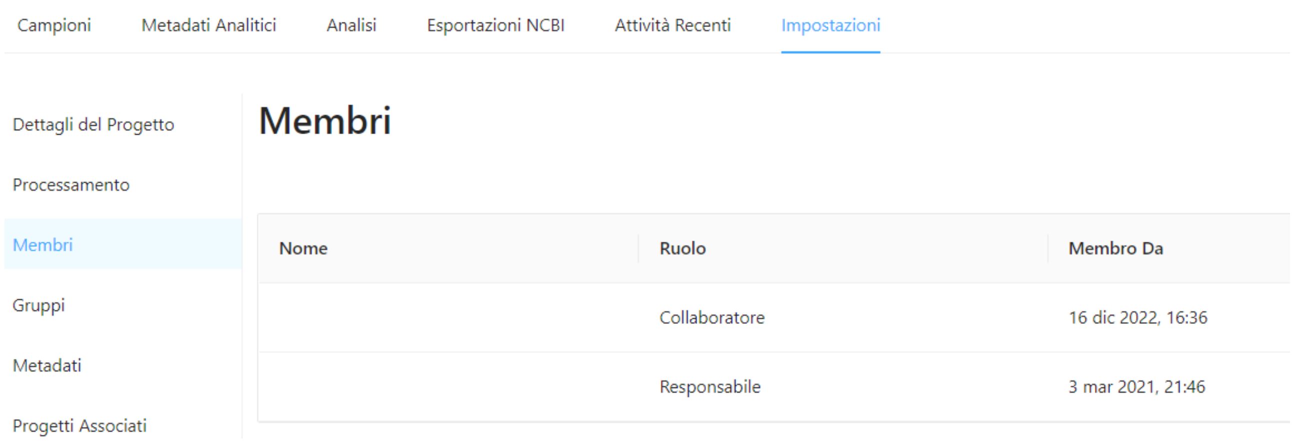

## Adding new samples to the Regional Project

Inserting a new sample into your project is divided into four operations:

1. Add a New Sample
2. Insert a Sample Name
3. Insert Sample Metadata (optional)
4. Upload sequence files fastq (IonTorrent single-end reads) or (Illumina paired-end reads)

Upon completion of the fastq file(s) upload, FastQC analysis for fastq sequence quality control and PHANtAsTiC analysis for sample assembly, typing and clustering or RECoVERY for mapping and characterization will be automatically performed.

A detailed description of the four aforementioned operations follows.

1. In the Regional Project from the menu **Azioni sui Campioni** (Actions on Samples) select **Aggiungi Campione Nuovo** (Add New Sample).

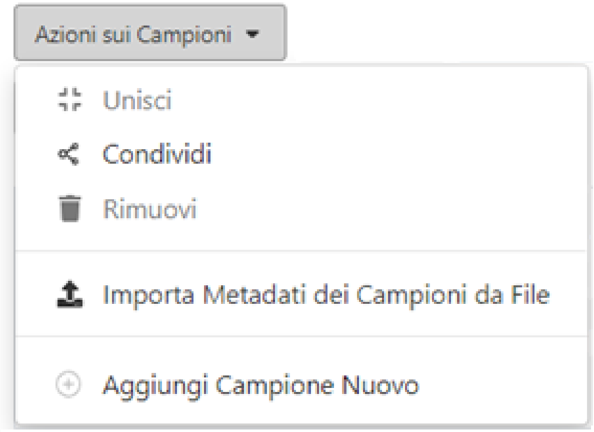


2. Insert a unique new name for the sample.

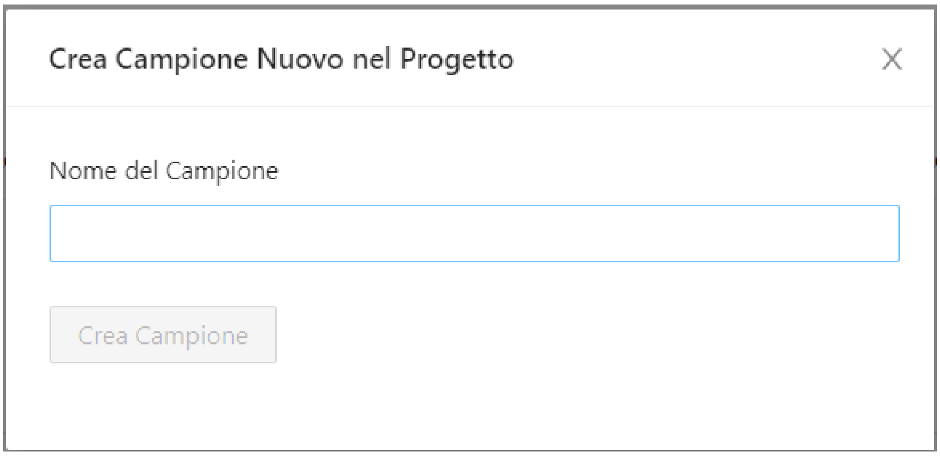

3. Edit the sample details in the section **Dettagli** (Details).

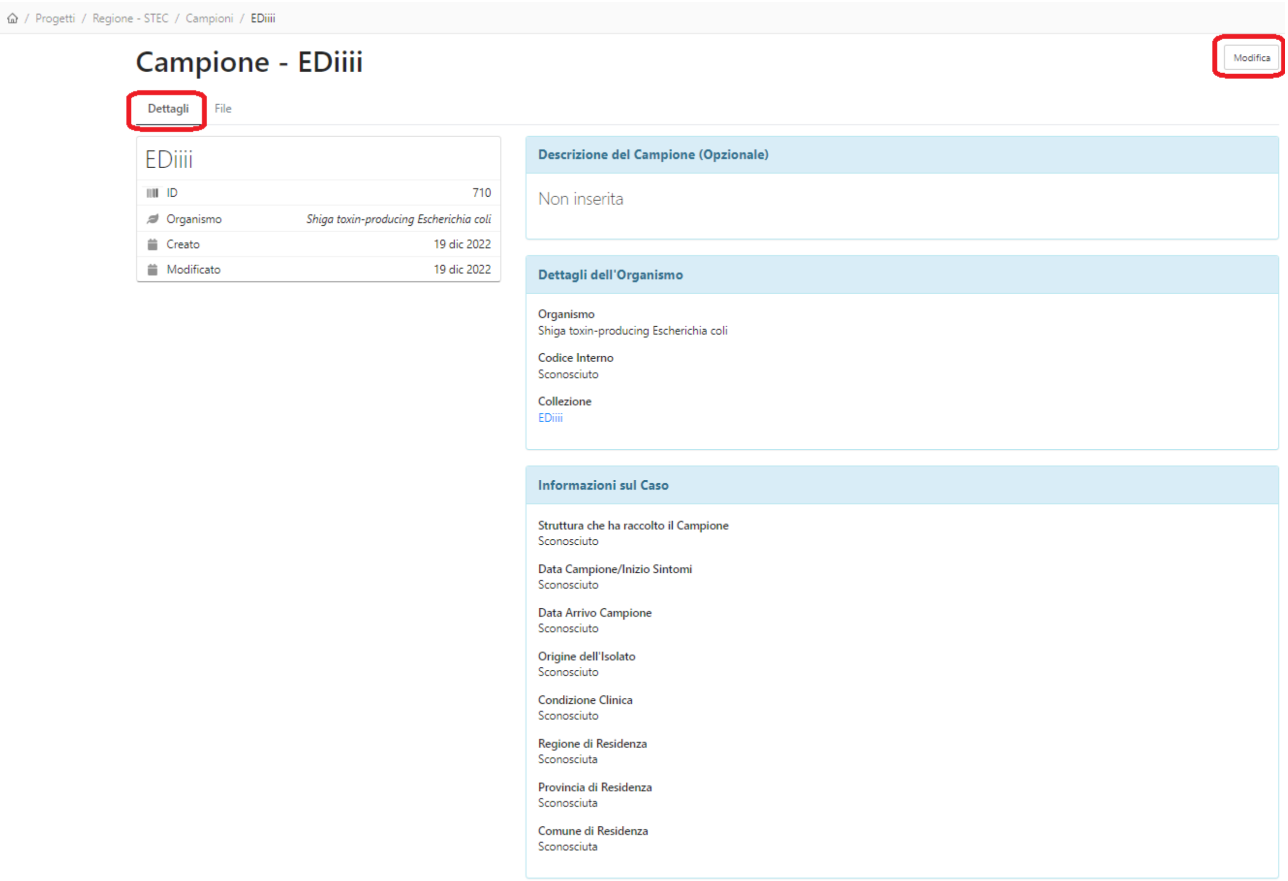


4. Upload sequence files in the section **File**, one file if single-end reads (IonTorrent), two files together if paired-end reads (Illumina)

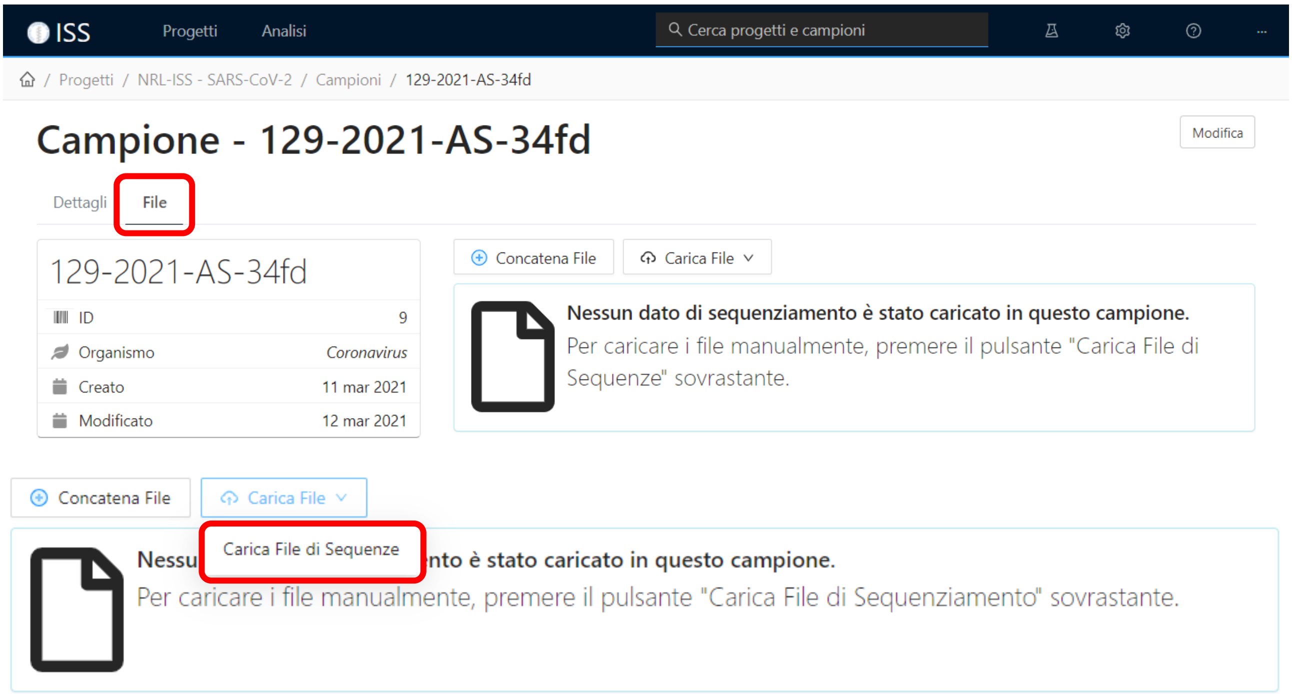

The page must not be left until the loading and initial processing are finished.

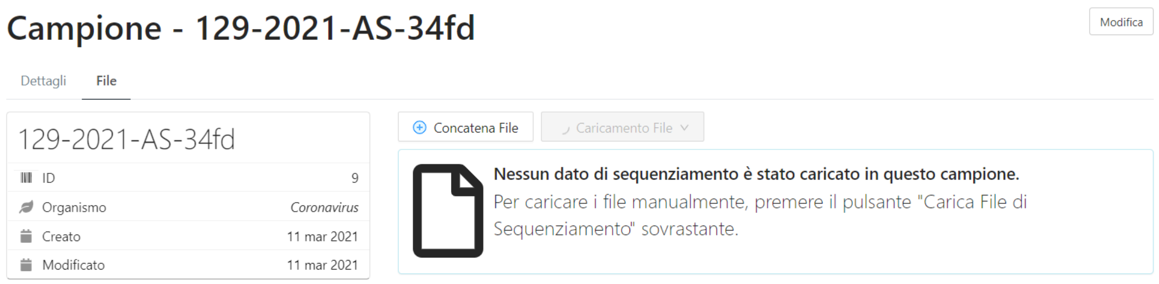


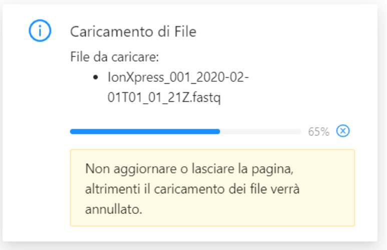


From the moment the processing state turns to **In esecuzione** (Executing), the page can be exited because processing will not stop even if the user exits the program or turns off the computer.

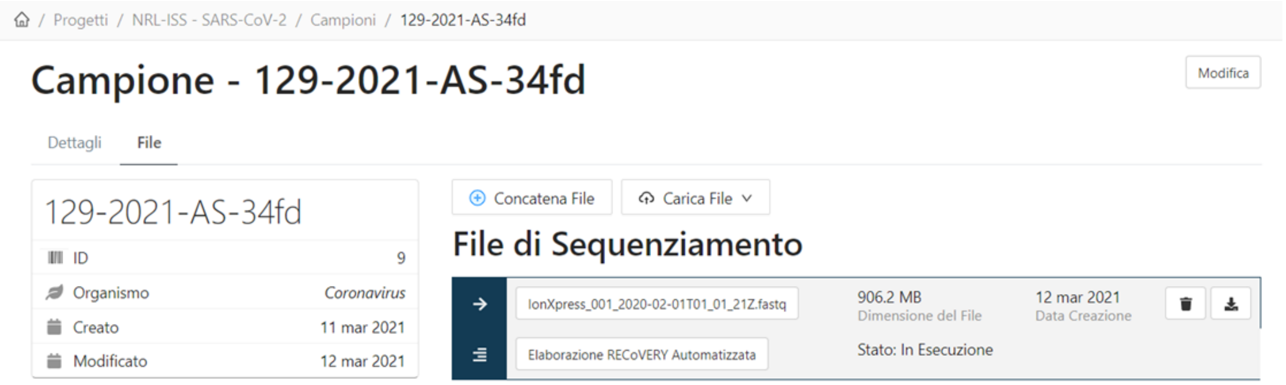


The FastQC quality analysis result can be inspected by clicking on the sequence file name. The page shows some values and graphs with the details of the surveys.

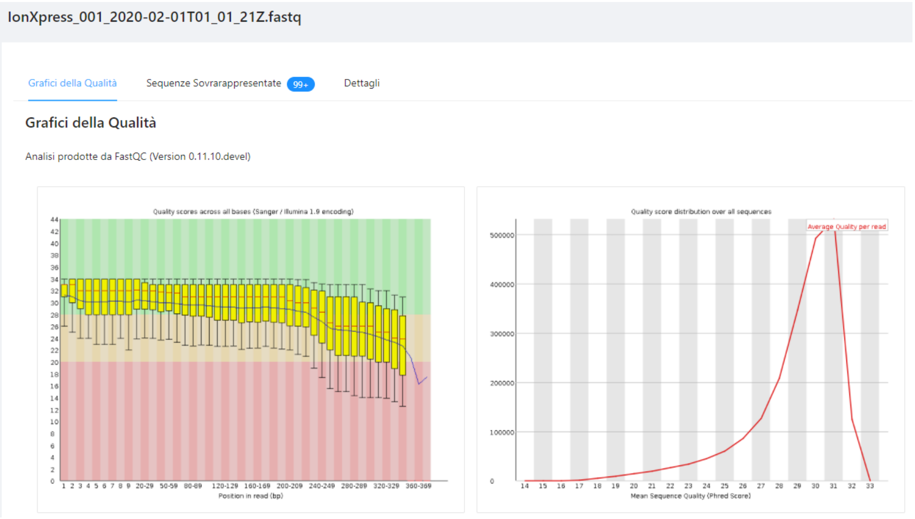

Upon completion of the automated processing, the Status changes to Completata (Completed). Furthermore, the system sends an end-of-analysis e-mail notification to all users of the Project in the Region.

Single-end reads.

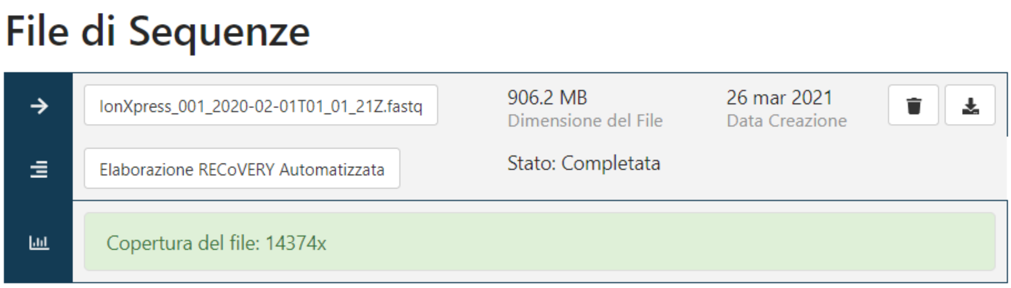


Paired-end reads.

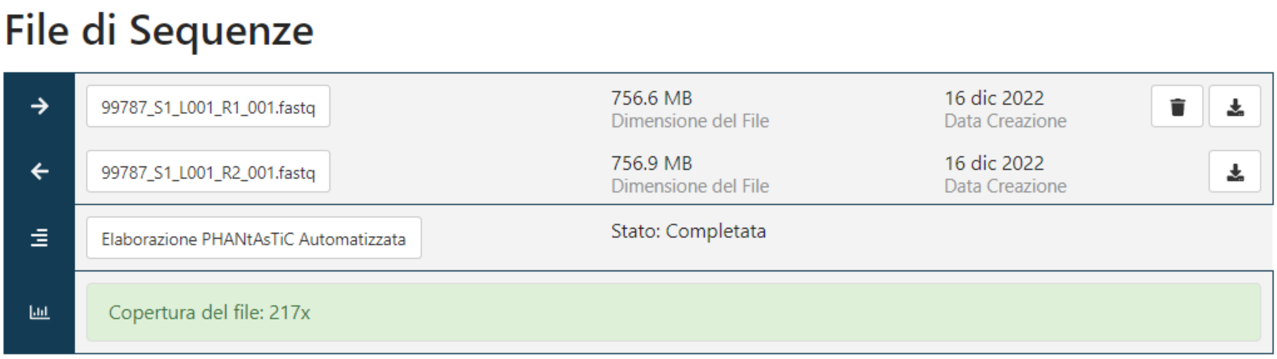


Returning to the section **Dettagli** (Details), the results of the analysis have now been entered in the **Altri Metadati** (Other Metadata) chapter. The Sample_Code is a unique value assigned by the system or the name of the sample and used in phylogenetic analysis. In the case of bacterial samples, the value of the Cluster_Id field will be "-" if it is not part of a cluster, otherwise the value will be "Cluster_" followed by the cluster number.

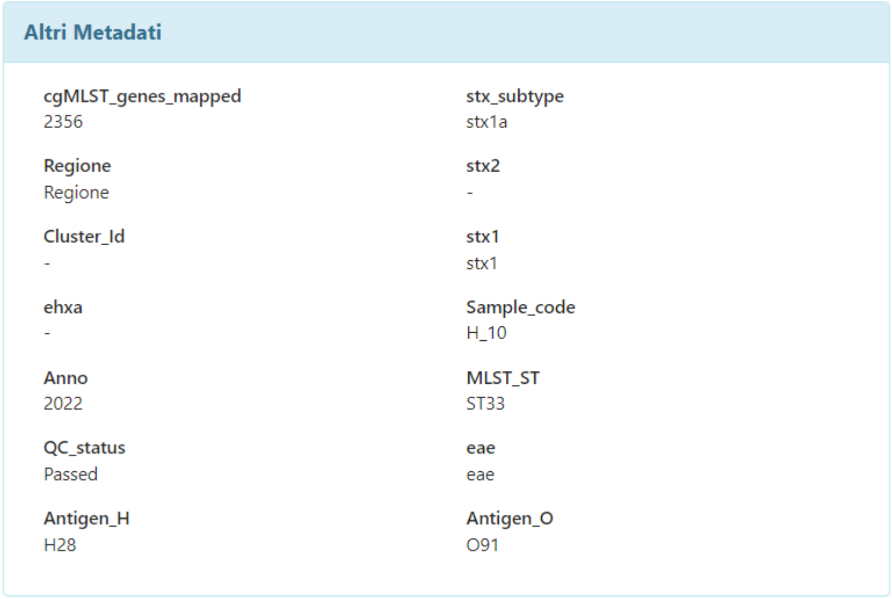

In the section **File**, selecting **Elaborazione [Nome Pipeline] Automatizzata** (Automated [Pieline Name] Analysis), the user is directed to the page that contains all the results of the analysis.

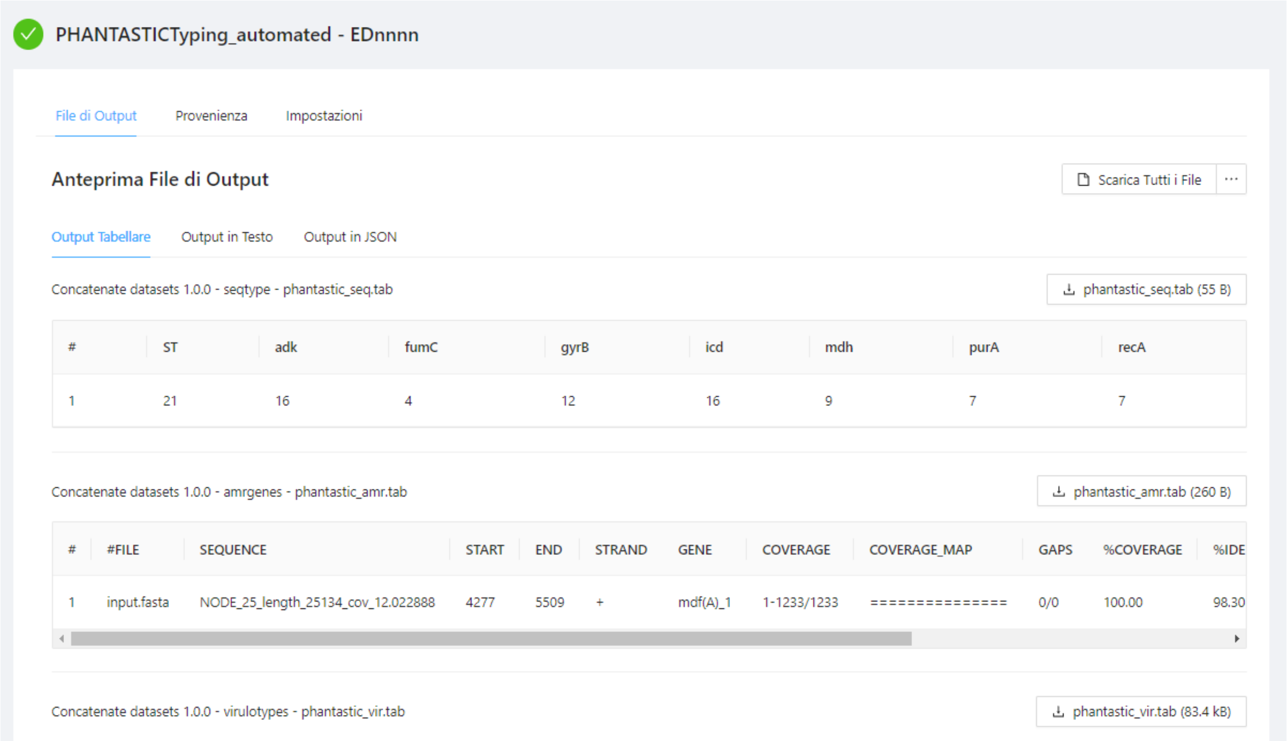


This page shows the details of the analysis and the output files that can be downloaded by the user. In the section **File di Output** (Output Files) a preview of the contents of the various output files is displayed.

# The Surveillance Project

From the Projects page, selecting the name Progetto Sorveglianza Genomica STEC or Listeria or I-Co-Gen, the national data of each genomica surveillance is accessed.

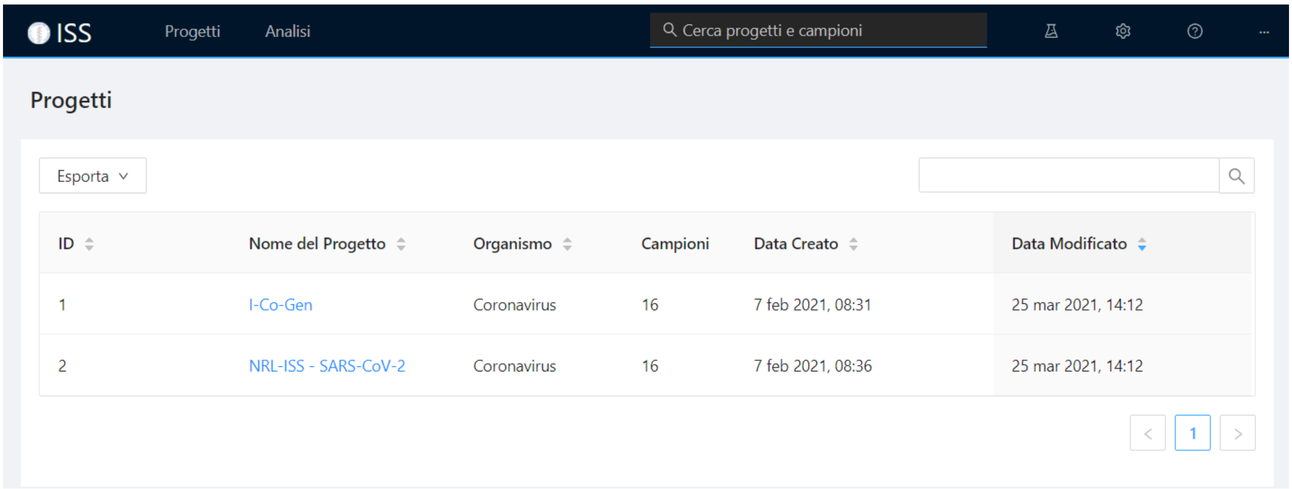


Compared to the Region's own Project page, some menus and items have been limited. Editing actions on samples are disabled. The operations permitted in this Project are the inspection of a restricted dataset of each sample and sample selection to send them to the Analysis Centre in the case of in-depth analysis.

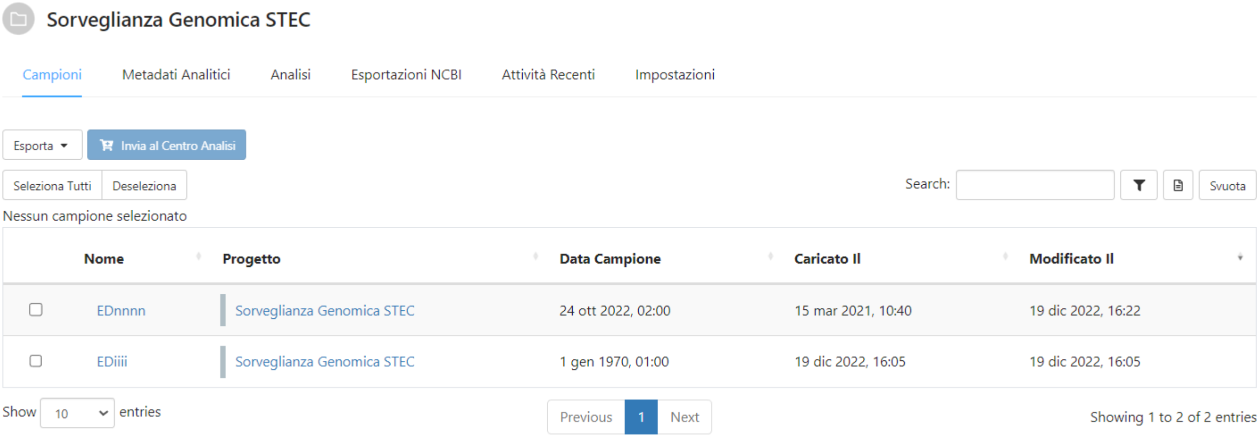


Any samples marked in red have quality problems with respect to the coverage of the file but have been included for surveillance purposes anyway.

# Analysis Centre

Clicking on the **Centro Analisi** (Analysis Center) icon on the ribbon at the top of each page will open the Analysis Center page. In this page it is possible to start analytical pipelines on the samples previously sent to it. The number of samples sent is displayed next to the icon.

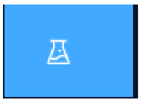

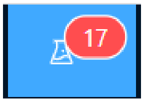


If there are samples selected, a sidebar opens where the samples sent to the Analysis Center are listed. There is the possibility of excluding individual samples or even all samples of a certain project by

selecting **Rimuovi Campione** (Remove Sample) next to the corresponding item. Furthermore, the Analysis Center can be completely emptied with the red **Svuota** (Empty) button at the bottom.

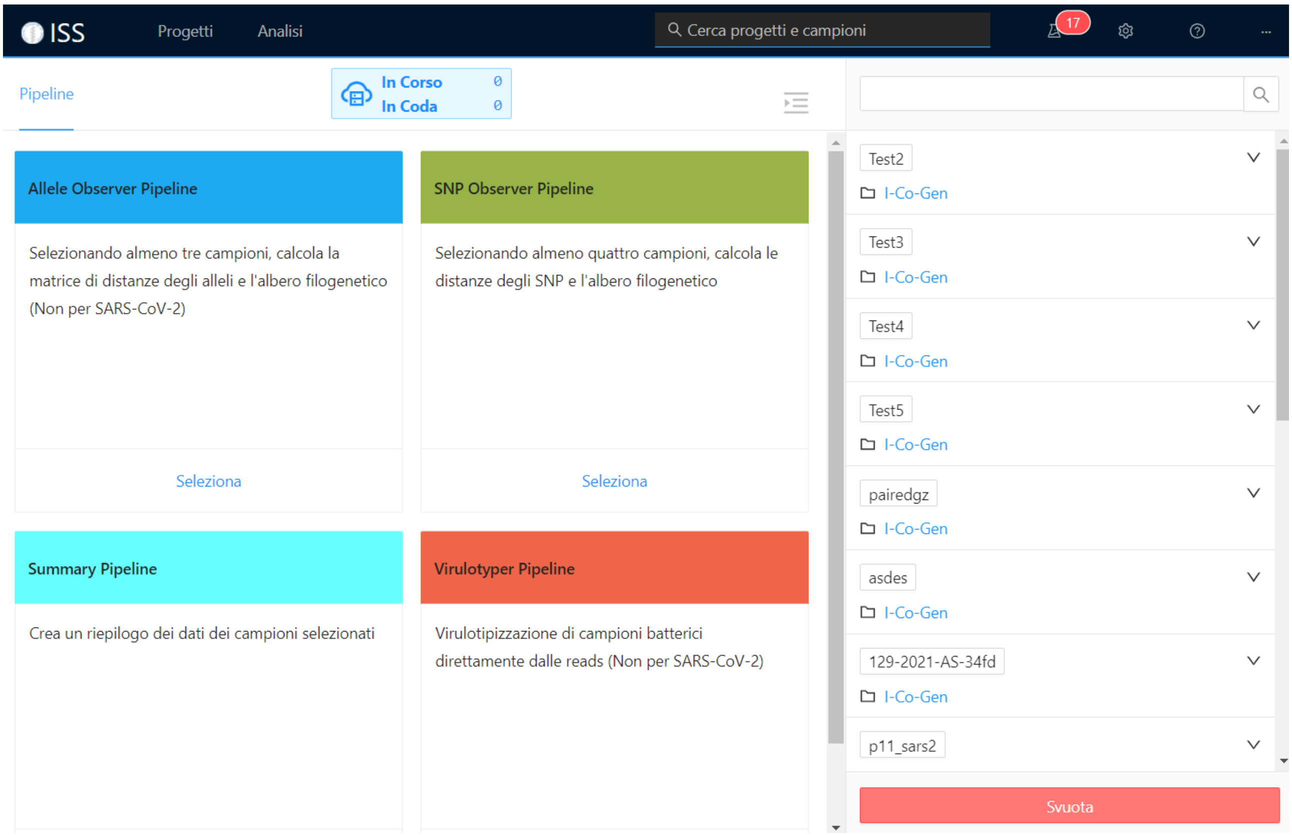

On the Analysis Center page it is possible to select the Analytical Pipelines to be performed on the selected samples.

The **Allele Observer Pipeline** (not for SARS-CoV-2) it is the same that is performed in the PHANtAsTiC processing of the samples. The pipeline is based upon cgMLST (core genome Multi Locus Sequence Type) allele calling of the sequences using chewBBACA (Article), then calculating the Hamming distance matrix between each sample and applying Neighbor joining to obtain a phylogenetic tree.

The **SNP Observer Pipeline** compare differences between sequences at the SNP level (Single Nucleotide Polymorphisms) instead of the allelic level with the tool PopPUNK (Article). Also in this case a phylogenetic tree is obtained by applying Neighbor joining on the distance matrix.

The **MST Observer Pipeline** calculates a Minimum Spanning Tree of cgMLST profiles and displays the result with metadata with GrapeTree (Article).

The **Esportazione Metadati Pipeline** creates a csv file with the selected samples' metadata.

The **Summary Pipeline** creates an html file with a summary of the data of the selected samples.

The **Virulotype Pipeline** (not for SARS-CoV-2) performs a typing of virulence genes by applying an optimized BLAST analysis (Articolo) on the sample.

The **Consensus Pipeline** (only for SARS-Cov-2) creates a multistep file with the consensus obtained from the RECoVERY analysis of own selected samples.

The **GISAID Pipeline** (only for SARS-Cov-2, maximum 1000 sequences) uploads the selected samples to the GISAID database.

Selecting a pipeline opens the launch page with all selected sample files listed. The user has the possibility to modify the Name of the Pipeline and/or to add a Description. The result of the analysis can be shared with the source project of the samples by ticking the appropriate box.

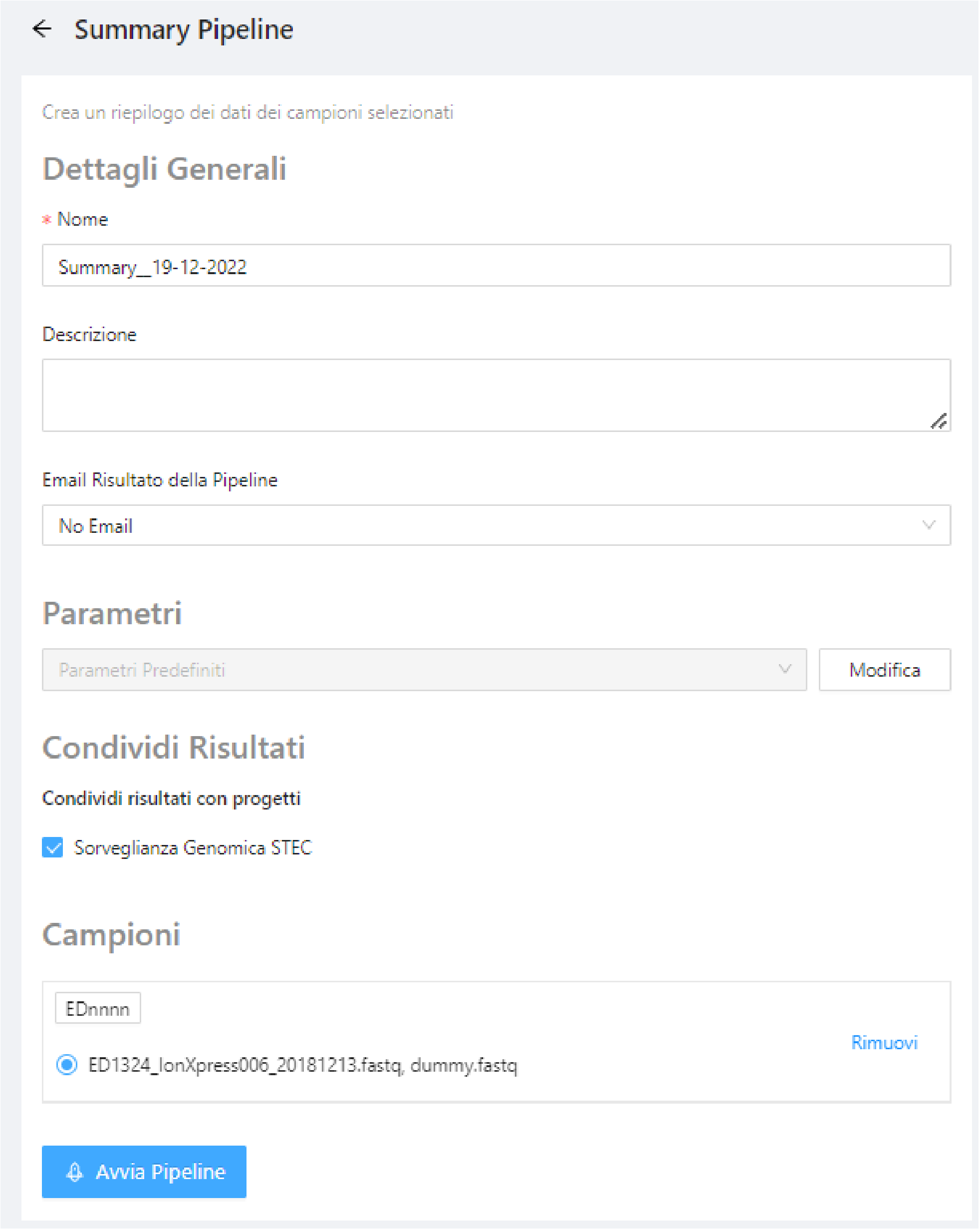


Selecting the button "Avvia pipeline" (Start pipeline), the pipeline is prepared for analysis and started.

Opening the menu **Analisi** (Analysis), the items **Analisi Proprie** (Own Analyses) and **File di Output** (Output Files) are shown.

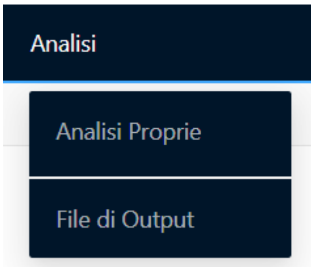

On the page **Analisi Proprie** (Own Analyses) all the analyses that the user has started are listed. The user can monitor the current status of the analyses here.

The consecutive phases of a pipeline run are: Queued, In Preparation, Prepared, Submitted, Starting, Executing, Finished Execution, In Completion, Transferring Results, Post Processing, Completed.

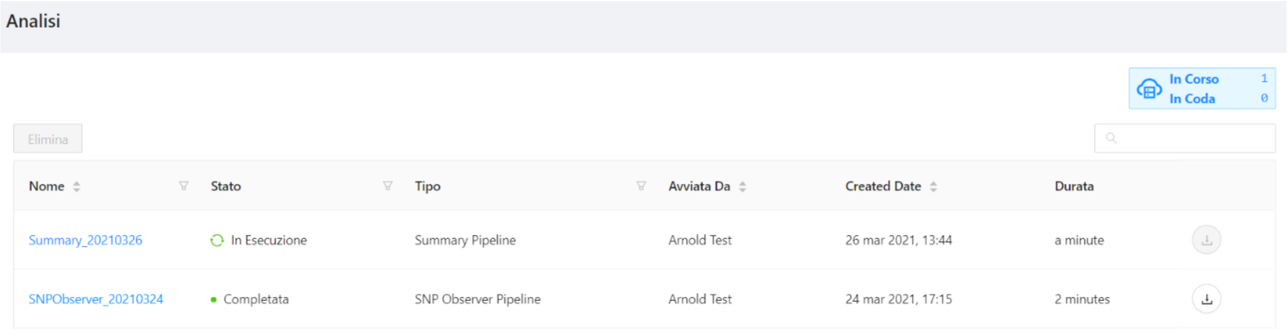


On the **File di Output** the files produced as a result of the analyzes that the user has performed are listed.

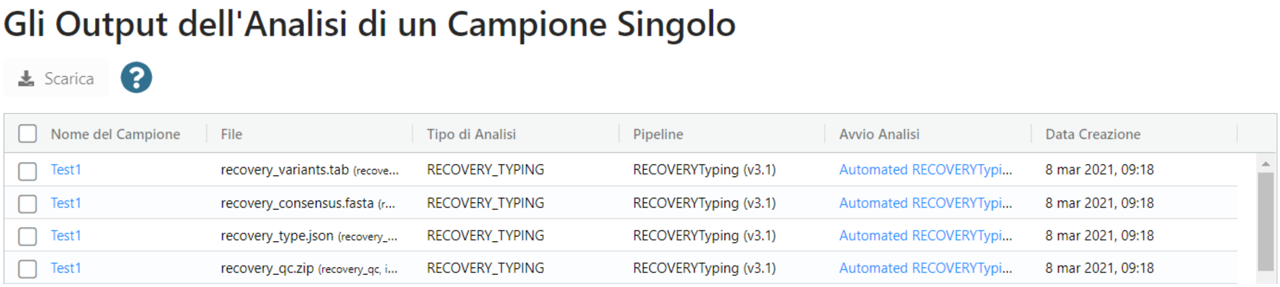

The result of the AlleleObserver and SNPObserver pipelines is a phylogenetic tree of the selected samples. For the result of both analyses, under the heading **Azioni** (Actions), there is the option **Mostra Visualizzazione Avanzata** (Show Advanced View).

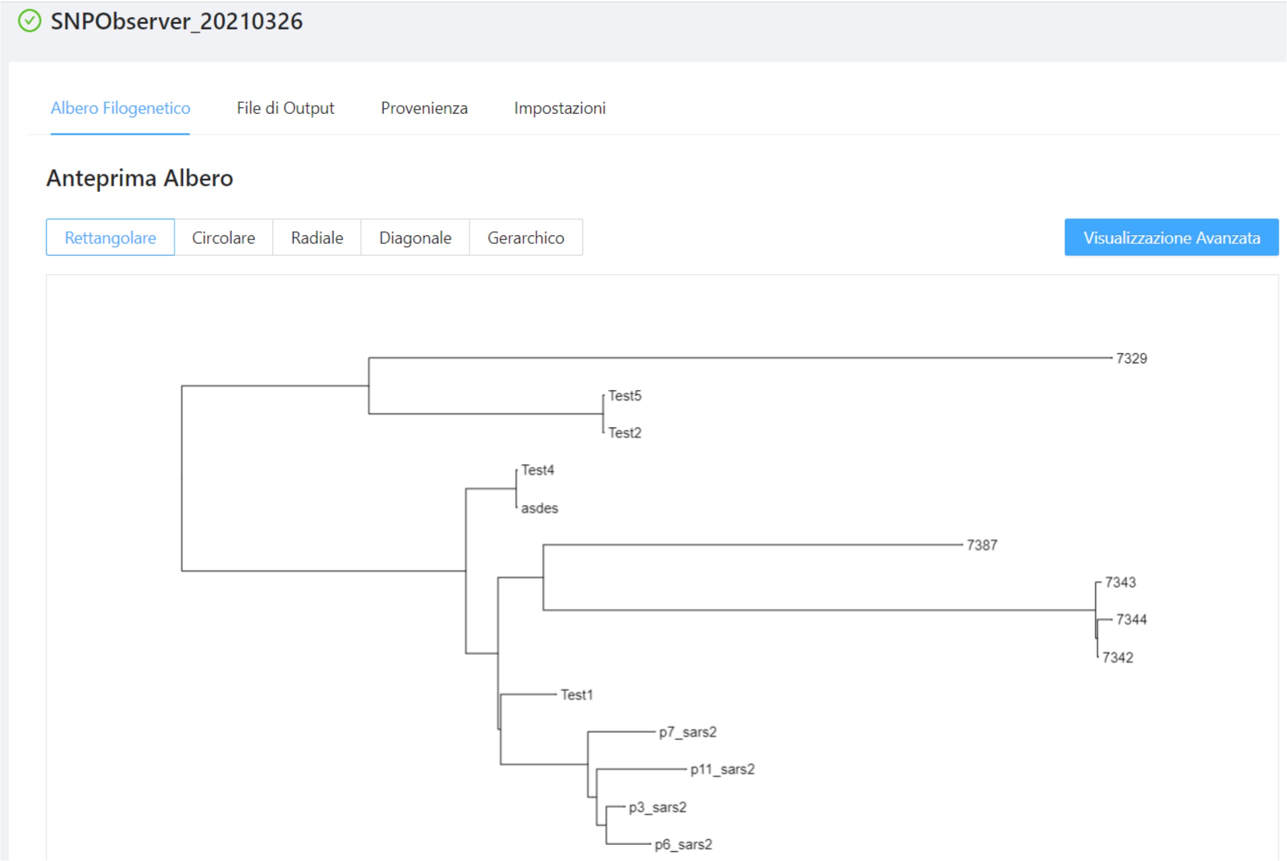


Clicking on the button opens a page with the phylogenetic tree again but this time with the metadata of each sample codified with a color side by side. With the **Alterna Metadati** (Toggle Metadata) button the metadata to be shown in the figure can be selected. The **Esporta SVG** (Export SVG) button allows to export the image in SVG format so as to be scalable. Please note that only part of the image visible in the browser window at that moment is exported.

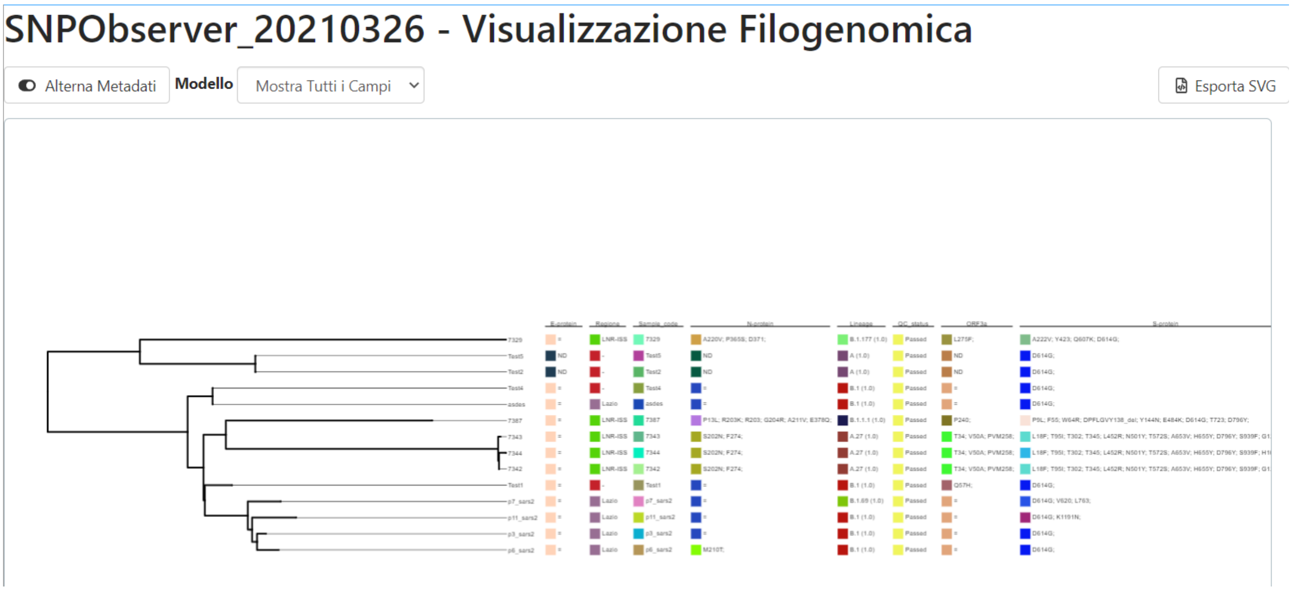

The MSTObserver pipeline instead also produces the visualization through the GrapeTree software accompanied by the metadata of the samples that can be used for contextualization.

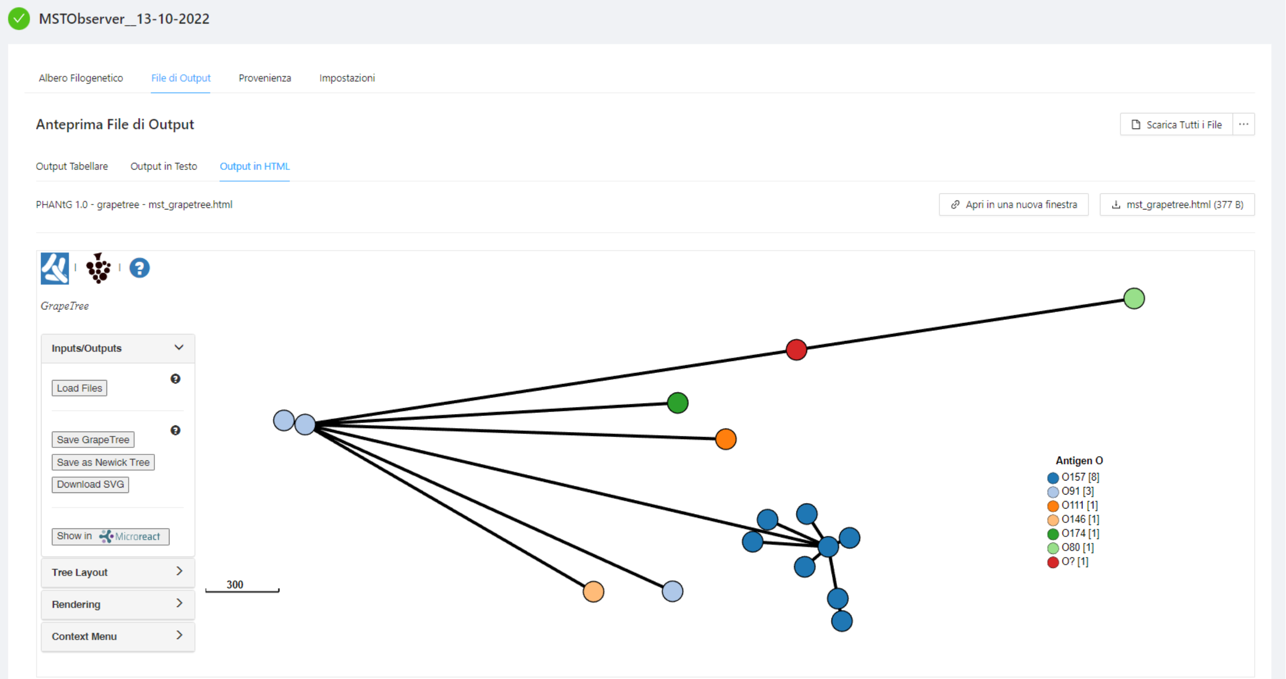


In the Summary pipeline, the result is an html file which is previewed but the iridaaries.html file can also be viewed in a new window or downloaded and viewed offline with any browser. The file contains a summary with various results referring to the samples under analysis.

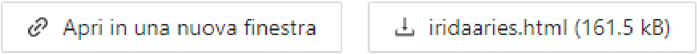


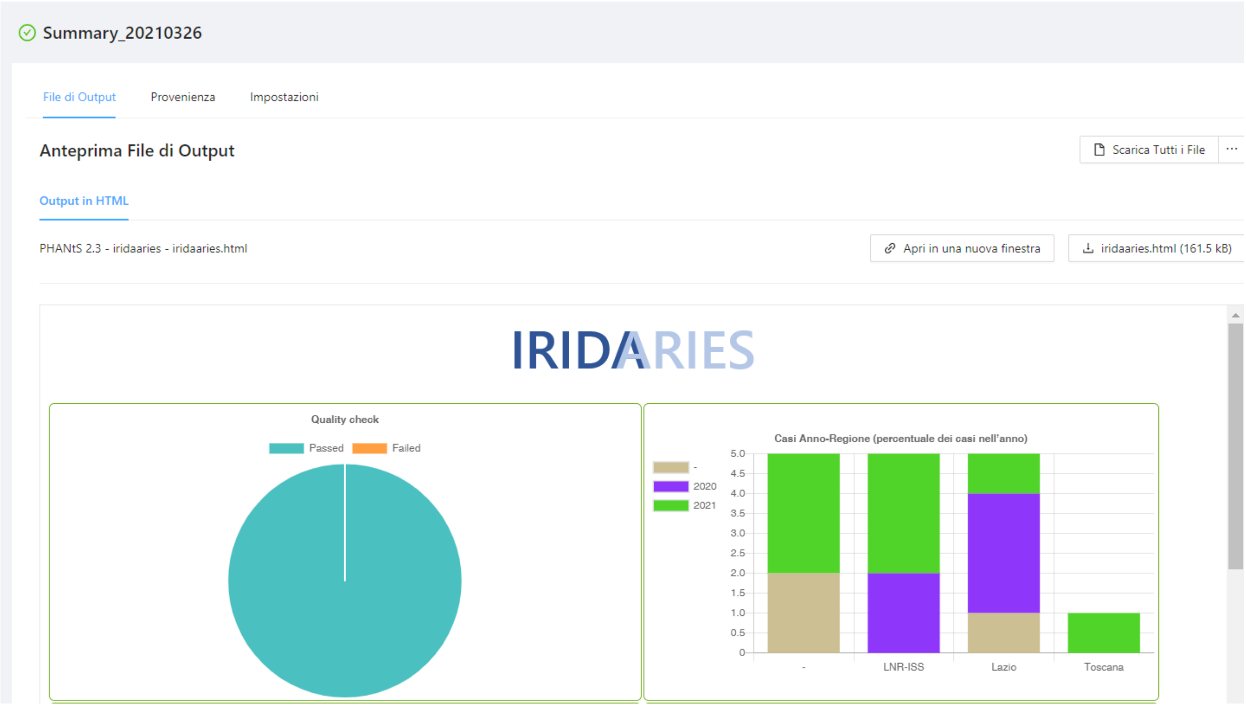

Supplement: Supplementary file 1 [file Data_Sheet_1.PDF]
